# Efficient near-infrared organic light-emitting diodes with emission from spin doublet excitons

**DOI:** 10.1038/s41566-024-01458-3

**Published:** 2024-06-19

**Authors:** Hwan-Hee Cho, Sebastian Gorgon, Giacomo Londi, Samuele Giannini, Changsoon Cho, Pratyush Ghosh, Claire Tonnelé, David Casanova, Yoann Olivier, Tomi K. Baikie, Feng Li, David Beljonne, Neil C. Greenham, Richard H. Friend, Emrys W. Evans

**Affiliations:** 1 Cavendish Laboratory, University of Cambridge, Cambridge, UK; 2 Laboratory for Computational Modelling of Functional Materials, Namur Institute of Structured Matter, University of Namur, Namur, Belgium; 3 Laboratory for Chemistry of Novel Materials, University of Mons, Mons, Belgium; 4 Department of Materials Science and Engineering, Pohang University of Science and Technology (POSTECH), Pohang, Republic of Korea; 5 Institute for Convergence Research and Education in Advanced Technology, Yonsei University, Seoul, Republic of Korea; 6 Donostia International Physics Centre, Donostia, Spain; 7 Ikerbasque Foundation for Science, Bilbao, Spain; 8 State Key Laboratory of Supramolecular Structure and Materials, College of Chemistry, Jilin University, Changchun, China; 9 Department of Chemistry, Swansea University, Swansea, UK; 10 Centre for Integrative Semiconductor Materials, Swansea University, Swansea, UK; 11 Present Address: Department of Chemistry and Industrial Chemistry, University of Pisa, Pisa, Italy; 12 Present Address: Institute of Chemistry of OrganoMetallic Compounds, National Research Council (ICCOM-CNR), Pisa, Italy

**Keywords:** Organic LEDs, Photochemistry

## Abstract

The development of luminescent organic radicals has resulted in materials with excellent optical properties for near-infrared emission. Applications of light generation in this range span from bioimaging to surveillance. Although the unpaired electron arrangements of radicals enable efficient radiative transitions within the doublet-spin manifold in organic light-emitting diodes, their performance is limited by non-radiative pathways introduced in electroluminescence. Here we present a host–guest design for organic light-emitting diodes that exploits energy transfer with up to 9.6% external quantum efficiency for 800 nm emission. The tris(2,4,6-trichlorophenyl)methyl-triphenyl-amine radical guest is energy-matched to the triplet state in a charge-transporting anthracene-derivative host. We show from optical spectroscopy and quantum-chemical modelling that reversible host–guest triplet–doublet energy transfer allows efficient harvesting of host triplet excitons.

## Abstract (web summary)

Exploiting the energy transfer between the host triplet states and spin doublet exciton states of a radical organic emitter enables near-infrared organic light-emitting diodes with an external quantum efficiency up to 9.6% at an emission wavelength of 800 nm.

## Main

Advances in efficient near-infrared (NIR) organic light-emitting diodes (OLEDs) can enable light generation in the biological window for healthcare diagnosis and treatment. The requirement for long-wavelength light generation beyond the visible range is also motivated by communications and security applications. Although an external quantum efficiency (EQE) of >20% in electroluminescence (EL) has been demonstrated for visible-light OLEDs, and commercial displays are commonplace, the performance of NIR OLEDs is generally limited to 5% EQE using fully organic emitters with emission peak wavelengths at 800 nm and longer^1^. The materials approach and mechanisms for efficient visible-light OLEDs by maximizing luminescence from singlet and triplet excitons have not translated to efficient NIR OLEDs.

Doublet fluorescence from organic radicals is an emerging basis for highly efficient NIR light-emitting devices that exploit favourable optical, electronic and spin properties for optoelectronics^2–17^. Luminescent organic radicals can have high photoluminescence quantum yield (PLQY) in the NIR range, where immunity from normal ‘energy gap law’ considerations is enabled by suppressing the non-radiative losses through decoupling high-frequency vibrational modes^18^. Almost 100% internal quantum efficiency for EL was demonstrated in radical OLEDs exploiting tris(2,4,6-trichlorophenyl)methyl (TTM)-based radicals^4^. This performance shows that using the doublet-spin manifold in radicals for luminescence can circumvent the typical efficiency limits (25% internal quantum efficiency) arising from the formation of singlet and triplet excitons in standard closed-shell molecule-based devices^16,17^. We recently reported efficient NIR OLEDs from triphenyl-amine (TPA)-substituted (2-chloro-3-pyridyl)bis(2,4,6-trichlorophenyl)methyl with a maximum EQE of 6.4% for 800 nm peak emission^19^. Two-component host systems of electron and hole transport materials were used to tune charge mobility to counter unbalanced electron and hole currents that otherwise result in high efficiency roll-off at high current densities for radical OLEDs. This concept was later demonstrated using a single-component thermally activated delayed fluorescence host material that supports both efficient electron and hole transport simultaneously^20^. Energy transfer mechanisms using thermally activated delayed fluorescence materials for charge recombination and sensitization of radical emitters for EL show promise for high performance by moving the exciton generation event away from radicals^21^. Although the relatively small ∆*E*_ST_ < 0.1 eV in these designs will lead to high efficiency loss due to host exciton decay from the energy gap law as the devices are pushed to the performance limits for NIR emission.

Here we use an anthracene derivative, 2-methyl-9,10-bis(naphthalene-2-yl)anthracene (MADN), as an energy transfer host that combines with a TPA-substituted TTM (TTM-TPA) NIR radical emitter (see ref. ^18^ for synthetic details). MADN enables efficient charge transport to generate excitons that then transfer to TTM-TPA for doublet EL. The high-energy singlet state (near 3 eV) mitigates losses from the energy gap law, whereas its low-energy triplet state is spin-protected from non-radiative decay. The energy degeneracy between the MADN triplet and emissive TTM-TPA doublet state enables spin-allowed transfer and efficient delayed emission. A maximum EQE for OLEDs of 9.6% is obtained at ∼800 nm with reduced efficiency roll-off, enhanced radiance and device stability.

## Results and discussion

### Near-infrared radical design of intersystem energy transfer

**Fig. 1:**
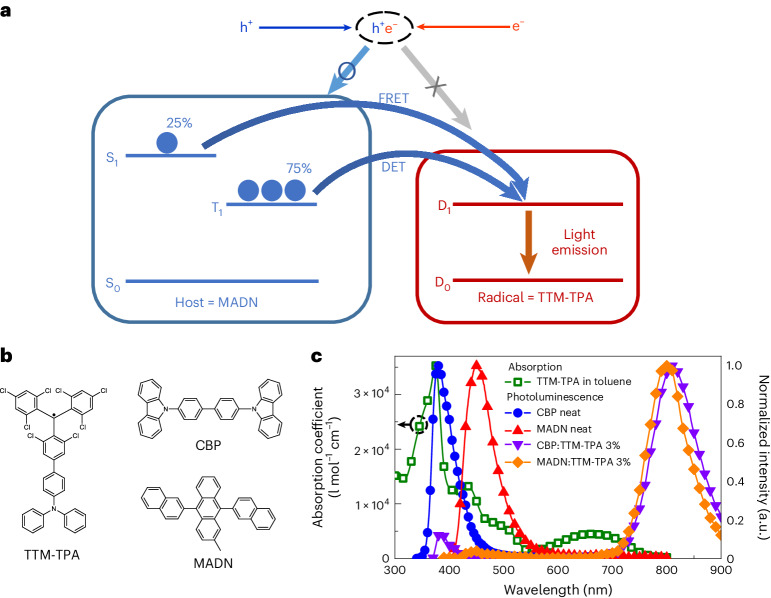
Radical energy harvesting design for high-efficiency NIR emission. **a**, Schematic illustration of intersystem dual energy transfer between host MADN and radical TTM-TPA in doublet EL devices. DET, Dexter energy transfer. **b**, Chemical structures of TTM-TPA, CBP and MADN. **c**, Absorption coefficient for TTM-TPA in toluene, as well as film PL spectra of CBP neat, MADN neat and TTM-TPA 3% doped in CBP and MADN. The spectral overlap between TTM-TPA absorption (open green squares) with CBP (filled blue circles) and MADN (filled red triangles) PL allows singlet–doublet energy transfer.

Figure 1a shows the available energy transfer pathways between singlet (S_1_) and triplet (T_1_) excitons of MADN, and doublet (D_1_) excitons of TTM-TPA in the MADN:TTM-TPA system (see Fig. 1b for the chemical structures). The scheme demonstrates the potential for energy harvesting of both singlet and triplet excitations in the non-radical host to form radical dopant states. This strategy exploits efficient spin-conserving transfer processes in the two pathways: singlet–doublet Förster resonance energy transfer (FRET) (S_1_ + D_0_ → S_0_ + D_1_) and triplet–doublet Dexter energy transfer (T_1_ + D_0_ → S_0_ + D_1_). MADN triplet emission extends between 700 nm and 900 nm (ref. ^22^), which is energy-resonant with TTM-TPA doublet emission (Fig. 1c). Accordingly, the MADN:TTM-TPA system enables the study of exciton harvesting in the limit of the small energy difference (|*∆E*_TD_| < 0.1 eV) between MADN T_1_ and TTM-TPA D_1_, where substantial host non-radiative losses due to the energy gap law are minimized.

The steady-state photophysical properties of TTM-TPA, MADN and 4,4’-bis(carbazol-9-yl)biphenyl (CBP) are depicted in Fig. 1c. Steady-state photophysical characteristics of TTM-TPA in different solutions are shown in Supplementary Fig. 1. The variation in the photoluminescence (PL) peak wavelengths at ~730~890 nm with an increase in solvent polarity suggests the formation of intramolecular charge-transfer excited states, in line with our previous reports^4,19^. The CBP:TTM-TPA system is used as a reference for studies of energy transfer mechanisms in the MADN:TTM-TPA system. The TTM-TPA-doped CBP and MADN films were made by a vacuum deposition process. TTM-TPA is sublimed between 180 °C and 200 °C for 3% doping under high vacuum (<5 × 10^−7^ Torr)—much lower than its decomposition temperature (345 °C)^18^. The NIR emission from TTM-TPA in films is matched to the PL in solution (Supplementary Fig. 1), supporting the high thermal stability of TTM-TPA. The PL spectra of the MADN and CBP neat films overlap with the absorption of TTM-TPA so that photoexcited singlet excitons generated in the host non-radical components in MADN:TTM-TPA and CBP:TTM-TPA systems (at 330 nm and 370 nm for CBP and MADN, respectively; see Supplementary Fig. 2 for absorption spectra for CBP and MADN neat films) undergo efficient singlet–doublet transfer to the radical guest, with NIR fluorescence observed at ~800 nm (Fig. 1c). Small contributions of host emission to the total PL are observed and provide characteristic signatures for the singlet–doublet energy transfer channels in these systems. The PLQY of TTM-TPA in toluene is 24% (excited at 370 nm), whereas the CBP:TTM-TPA 3% and MADN:TTM-TPA 3% films have PLQYs of 19% and 27%, respectively, at the same host excitation wavelength. This shows that high-energy singlet materials with efficient singlet–doublet transfer can be used to host NIR radical emitters.

### High-performance NIR radical OLEDs

**Fig. 2:**
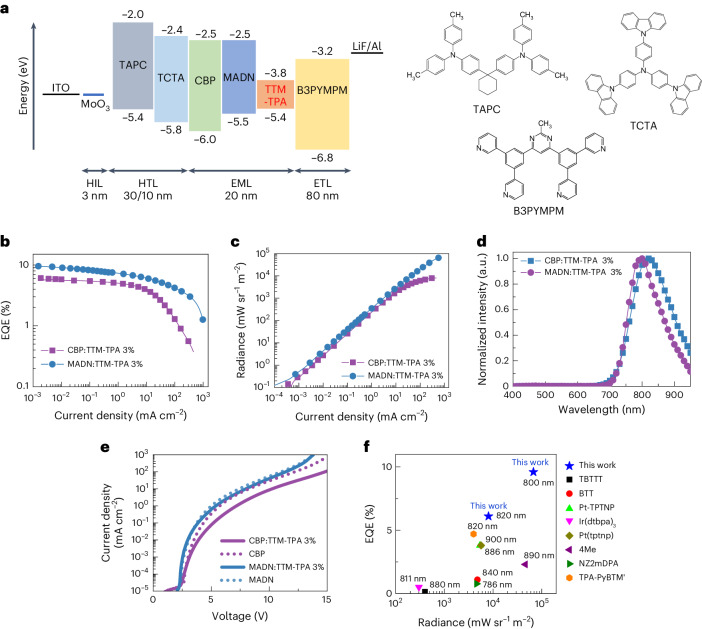
Radical OLED device structure and optoelectronic characterization. **a**, Device structure with energy levels for hole injection layer (HIL), hole transport layer (HTL), emitting layer (EML) and electron transport layer (ETL). TAPC, 1,1-bis[(di-4-tolylamino)phenyl]cyclohexane; TCTA, 4,4′,4″-tris(carbazol-9-yl)triphenyl-amine. **b**,**c**, EQE–current density (**b**) and radiance–current density (**c**) plots for the devices. **d**, EL spectra at 1 mA cm^−2^. **e**, *J*–*V* characteristics for the devices with and without TTM-TPA doping. **f**, Comparison of NIR OLEDs with peak wavelengths between ~780 nm and ~900 nm regarding maximum EQE and radiance (Supplementary Table 2).

The device structure of the radical OLEDs based on the TTM-TPA-doped emitting layer studied in this work is depicted in Fig. 2a. Using standard OLED design and vacuum deposition, hole injection from ITO/MoO_3_ was combined with hole transport layers of 1,1-bis[(di-4-tolylamino)phenyl]cyclohexane and 4,4′,4″-tris(carbazol-9-yl)triphenyl-amine. Electron injection was obtained from Al/LiF using a bis-4,6-(3,5-di-3-pyridyl phenyl)-2-methyl pyrimidine (B3PYMPM) electron transport layer. The device characteristics for the current density, voltage, EQE, radiance and EL profile are shown in Fig. 2b–e and summarized in Table 1. The resulting MADN:TTM-TPA OLED gives NIR EL at a peak wavelength of 800 nm with a maximum EQE of 9.6% (Fig. 2b), which is much higher than previous performance limits for reported NIR OLEDs beyond 780 nm peak emission^1,19^, and higher efficiency than reference devices using hosts of CBP (Fig. 2b) and CBP:B3PYMPM exciplex (Supplementary Fig. 4). Whereas the CBP:TTM-TPA device suffers a large efficiency drop beyond 10 mA cm^−2^, the MADN:TTM-TPA device sustains a relatively high efficiency of 4.2% up to 100 mA cm^−2^. Consequently, the maximum radiance of the MADN:TTM-TPA device reaches 68,000 mW sr^−1^ m^−2^, which is nearly an order of magnitude higher than the 8,100 mW sr^−1^ m^−2^ obtained in the CBP:TTM-TPA device (Fig. 2c).

**Table 1:** Summary of device performance

	*V*_on_^a^ (V)	EQE_Max_ (%)	EQE_*J*-1.0_^b^ (%)	EQE_*J*-100.0_^c^ (%)	Radiance_Max_ (mW sr^−1^ m^−2^)	*λ*_max_ (nm)
CBP:TTM-TPA 3%	2.8	6.1	4.9	1.3	8,100	820
MADN:TTM-TPA 3%	2.4	9.6	7.2	4.2	68,000	800

^a^Voltage at 10^−1^ mW sr^−1^ m^−2^.

^b^EQE at 1 mA cm^−2^.

^c^EQE at 100 mA cm^−2^.

Figure 2d shows the NIR doublet EL emission spectra for the devices. Interestingly, in contrast to the PL of CBP:TTM-TPA 3% and MADN:TTM-TPA 3% (Fig. 1c), no host emission is observed. This indicates that singlet–doublet energy transfer is not the main mechanism at play for EL. Current density–voltage (*J*–*V*) plots for devices with and without radical doping are shown in Fig. 2e. First, we observe a steeper *J*–*V* gradient for MADN:TTM-TPA versus CBP:TTM-TPA devices. This is consistent with MADN having better electron and hole transporting properties than CBP, as demonstrated using single-carrier device analysis (Supplementary Section 2). Second, we find that TTM-TPA doping causes negligible differences between *J*–*V* curves for MADN:TTM-TPA and MADN-only devices, whereas a much shallower curve is seen in CBP:TTM-TPA versus CBP-only devices. We consider that this indicates radical energy transfer through singlet and triplet channels following exciton formation at host MADN sites in the MADN:TTM-TPA device (Fig. 1a), whereas the *J*–*V* characteristics for the CBP-based device suggest the involvement of radical charge trapping^23^. We have tested these devices for stability under constant drive. These devices are exposed to the nitrogen atmosphere between sublimation steps and are operated without encapsulation. Under these conditions, we do find the MADN:TTM-TPA device shows nearly a tenfold-improved lifetime (to 50% EL) of 58 h (at 0.1 mA cm^−2^) compared with 7 h in the CBP:TTM-TPA device (Supplementary Fig. 5), which also presents a substantial increase over previous results^12,19^. The much better stability of the MADN:TTM-TPA device can be understood on the basis of the different electrical properties of the two hosts. As we see from single-carrier device analysis (Supplementary Section 2), the CBP:TTM-TPA emitting layer shows poor electron transporting properties but very high hole transporting properties, which leads to the narrow emission zone causing exciton quenching and low stability^20^. Furthermore, we consider the different emission mechanisms that operate in the two device types. The CBP:TTM-TPA device operates mainly via a charge-trapping-based emission mechanism, whereas efficient energy transfer from MADN to TTM-TPA is dominant in the MADN:TTM-TPA device, which will be further demonstrated by transient and magneto-electroluminescence (MEL) combined with transient photophysical studies below. Under charge-trapping-based EL processes, the high population of excitons concentrated and trapped at radicals is easily quenched, causing large efficiency roll-off and degradation. By contrast, energy-transfer-based EL processes lead to reduced exciton quenching and efficiency roll-off, which enables much better device stability. The MADN:TTM-TPA device performance sets a new benchmark for stability (which is not generally reported for NIR OLEDs)^1,24,25^, and also exhibits higher maximum efficiency and radiance than other reported ~780–900 nm devices, as summarized in Fig. 2f and Supplementary Table 2. Organic LEDs based on luminescent organic radicals represent a promising solution to close the efficiency gap between devices using fully organic NIR emitters and hybrid/inorganic systems.

**Fig. 3:**
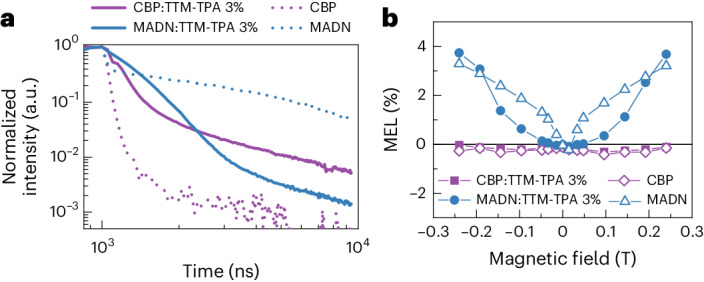
Transient EL and MEL studies of radical OLEDs. **a**, Transient EL profiles for CBP and MADN devices with and without TTM-TPA doping. The voltage pulse corresponds to 1 mA cm^−2^, and the off-voltage was −5 V for de-trapping the charge carriers after turn-off. No delayed emission is observed in the CBP-based devices (purple), whereas MADN-based devices (blue) show strong delayed emission features. **b**, Magneto-electroluminescence for devices studied at 1 mA cm^−2^. The CBP-based devices show negligible MEL, whereas the MADN-based devices show positive signatures that reflect energy transfer contributions to the EL mechanism.

The active role of energy transfer in the mechanism for high-performance radical OLEDs was explored by time-resolved optical studies of working devices. On removal of electrical excitation, the transient EL of the MADN-only device shows delayed emission (*τ* = 4.3 ± 0.1 µs) that is characteristic of singlet fluorescence following triplet–triplet annihilation (TTA) (blue dotted line, Fig. 3a)^26,27^. The CBP-only device shows no delayed emission in transient EL but only prompt decay (*τ* = 44 ± 1 ns), which suggests that triplets formed in CBP do not contribute to the overall emission process (purple dotted line, Fig. 3a). As expected from the full device and single-carrier device characteristics (Fig. 2b–e and Supplementary Section 2), the CBP:TTM-TPA OLED shows fast prompt doublet fluorescence (*τ* = 150 ± 3 ns) where the initial feature of additional emission at ~50–100 ns is attributed to recombination with the trapped charges remaining at the radical sites from the previous excitation pulse (purple solid line, Fig. 3a)^23,28–30^. In contrast, the MADN:TTM-TPA device exhibits a delayed EL decay of *τ* = 370 ± 5 ns (blue solid line, Fig. 3a), which is different from the transient EL profile of the MADN-only device. This supports that the EL mechanism in the MADN:TTM-TPA device involves energy transfer from triplet excitations to emissive radical doublet states without an intermediate TTA process.

Magneto-electroluminescence studies were conducted on the MADN:TTM-TPA and reference devices: MADN-only, CBP-only and CBP:TTM-TPA (Fig. 3b). These studies provide further insights into the EL mechanism by elucidating the effects of magnetic fields on luminescence yield of exciton states in devices^26,31–36^. Here, MEL is defined as ${\rm{MEL}}\left( \% \right)=\frac{{\rm{EL}}\left(B\right)-{\rm{EL}}(0)}{{\rm{EL}}(B)},$ where EL(*B*) and EL(0) represent the EL intensity in the presence and absence of a magnetic field, *B*, respectively. The CBP-based devices show almost negligible magnetic field dependence of the EL regardless of TTM-TPA doping in CBP-only (open purple diamonds) and CBP:TTM-TPA (filled purple squares) devices in Fig. 3b.

The MADN-based devices with and without TTM-TPA doping are distinguished from the CBP-based devices by positive MEL profiles. The net-positive MEL in the MADN-only OLED is attributed to magnetosensitivity of the polaron-pair hyperfine mechanism (positive MEL) that dominates over the dependence from TTA (negative MEL)^26,31,36^. The non-identical MEL profiles for the MADN:TTM-TPA device compared to the MADN-only device also imply an EL mechanism without indirect radical energy harvesting by TTA. The broader magnetic field dependence in the MEL profile for the MADN:TTM-TPA OLED is assigned to triplet–doublet energy transfer, where magnetosensitivity originates from larger triplet zero-field splitting interactions (>10 mT) compared with smaller hyperfine interactions (~1–10 mT) in the polaron-pair mechanism^31,34^.

### Exciton dynamics and energy transfer

**Fig. 4:**
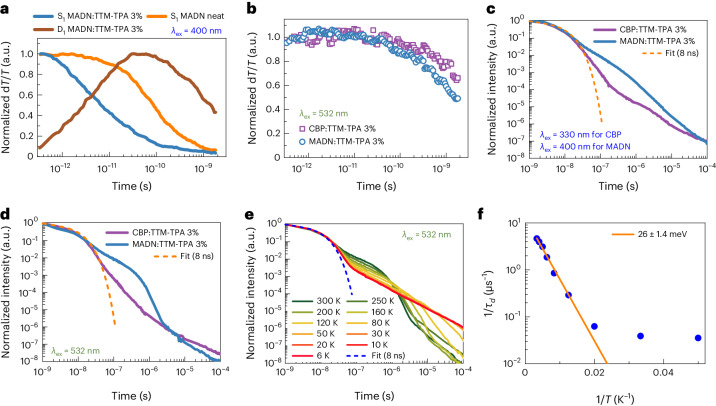
Time-resolved spectroscopy. **a**, Excited-state singlet (S_1_) and doublet (D_1_) population kinetics extracted from transient absorption of neat MADN and MADN:TTM-TPA 3% films under 400 nm excitation. The decay of S_1_ in the blend and the matching rise of D_1_ are due to rapid singlet–doublet FRET. **b**, Comparison of D_1_ population kinetics for CBP:TTM-TPA 3% and MADN:TTM-TPA 3% films under radical-only 532 nm excitation. Faster decay observed in the MADN blend is indicative of doublet–triplet energy transfer. **c**,**d**, Transient PL profiles averaged over 720–880 nm for radical emission following host-selective (400 nm, 330 nm; **c**) and radical-selective (532 nm; **d**) excitation. Delayed radical emission is observed in the MADN blend under both host and radical excitation. **e**, Temperature-dependent transient PL profiles of MADN:TTM-TPA 3% excited at 532 nm. Delayed radical emission is faster at elevated temperatures. **f**, Arrhenius plot for the MADN:TTM-TPA 3% system revealing a small activation energy for delayed radical emission.

We performed transient optical spectroscopy studies to investigate the exciton dynamics and the available radical energy transfer pathways depending on T_1_–D_1_ energy alignment in these host–radical systems. Picosecond transient absorption studies were performed at low fluences to exclude exciton–exciton annihilation. Transient absorption under a 400 nm pump for host-selective excitation (Fig. 4a) reveals faster decay of MADN excited-state features assigned to S_1_ excitons in 3% TTM-TPA in MADN films compared with pristine MADN (Supplementary Fig. 12). The S_1_ decay for MADN:TTM-TPA mirrors a rise in D_1_-photoinduced absorption from the TTM-TPA component, where the timescale for singlet–doublet transfer is rapid (*τ*_SD_ = 8 ps). We establish that the picosecond dynamics of TTM-TPA is independent of excitation wavelength within 330–610 nm (Supplementary Fig. 13). Transient absorption studies under radical-selective photoexcitation were performed on CBP:TTM-TPA and MADN:TTM-TPA films with 532 nm excitation (Fig. 4b). Faster decay of D_1_ excitons is observed in MADN:TTM-TPA, where |∆*E*_TD_| < 0.1 eV, versus CBP:TTM-TPA, where |∆*E*_TD_| > 0.8 eV (CBP T_1_ = 2.6 eV)^37^. This suggests that radical D_1_ excitons formed via S_1_ → D_1_ FRET can transfer energy to closely lying excited triplet T_1_ states on MADN.

Transient PL studies were conducted on these host–radical films (Fig. 4c,d). Delayed TTM-TPA radical emission is observed in MADN:TTM-TPA film following both host- (400 nm; Fig. 4c) and radical-selective (532 nm; Fig. 4d) photoexcitation. The emission lineshape is unchanged throughout the decay (Supplementary Fig. 15). By contrast, in CBP:TTM-TPA film, the delayed component contributes less than 1% of all emitted photons under either excitation condition (Supplementary Fig. 16).

The transient absorption and PL dynamics in MADN:TTM-TPA film with low ∆*E*_TD_ allow us to conclude that triplet–doublet and doublet–triplet energy transfer pathways are present in this system (Supplementary Section 3). This results in excited-state (re)cycling of triplet and doublet states in a delayed emission mechanism. The temperature dependence of transient PL in MADN:TTM-TPA film shows thermal activation of delayed radical emission under selective radical excitation (Fig. 4e), where the only available processes are doublet luminescence, doublet–triplet and triplet–doublet energy transfer and triplet diffusion. Doublet luminescence is temperature-independent in donor–acceptor TTM radicals^38^. Arrhenius analysis reveals an activation energy of 26.0 ± 1.4 meV (Fig. 4f). This small energy gap is comparable with thermal energy (*k*_B_*T*) at room temperature and can therefore be efficiently overcome in OLEDs. We assign its origin to diffusion-limited reformation of triplet–radical encounter pairs, as described below.

### Modelling of energy transfer

An amorphous sample comprising MADN as the host doped with radical TTM-TPA molecules at a 3.1% m/m concentration was prepared using classical force-field molecular dynamics simulations. After equilibration, a few interacting MADN:TTM-TPA molecular complexes were extracted from the sample and their ground-state equilibrium geometries were then relaxed at the density functional theory (DFT) level (ωB97X-D/6-31G(d,p)). The structures of the selected complexes are shown in Supplementary Fig. 20. Vertical excitation energies were computed by resorting to an optimally tuned screened range-separated hybrid (OT-SRSH) approach (LC-ωhPBE/6-311G(d,p)) within the time-dependent DFT in the Tamm–Dancoff approximation (TDA) (Supplementary Section 4)^39,40^.

**Fig. 5:**
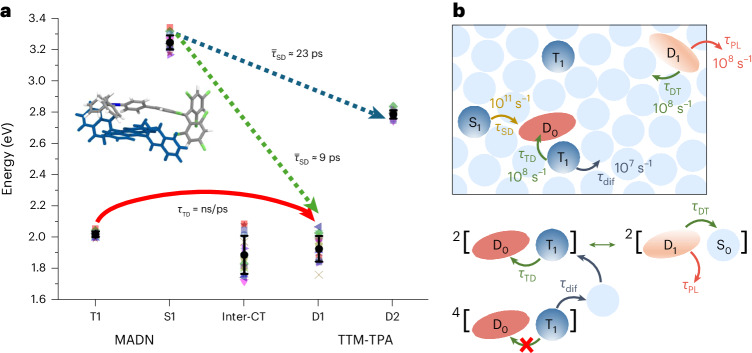
Excited-state pathways. **a**, Calculated energetic landscape for a range of 17 molecular complexes of MADN and TTM-TPA. The filled black circles represent the average excitation energies and vertical bars quantify the standard deviation: T_1_ = 2.02 ± 0.02 eV; S_1_ = 3.25 ± 0.04 eV; D_1_ = 1.93 ± 0.08 eV; D_2_ = 2.79 ± 0.03 eV; inter-^2^CT = 1.89 ± 0.12 eV. The inset shows the molecular conformation of **CP1**. The computed average lifetime of *τ*_SD_ to the D_2_ and D_1_ states is 23 ps and 9 ps, respectively. Energy transfer from T_1_ to D_1_ occurs in a superexchange-like mechanism mediated by the presence of low-lying inter-^2^CT states, with a computed overall lifetime *τ*_TD_ spanning from tens of nanoseconds to tens of picoseconds. **b**, Scheme of exciton pathways and their approximate rates. Triplet excitons form either an overall doublet (^2^[D_0_–T_1_]) or quartet (^4^[D_0_–T_1_]) encounter pair when adjacent to a radical site. Reversible energy transfer occurs in the doublet configuration, whereas quartet pairs separate during triplet diffusion.

In the most stable pair (labelled **CP1**), these calculations yield the first singlet (S_1_) and triplet (T_1_) excited states localized on the anthracene core of MADN at 3.17 eV and 2.00 eV, respectively, whereas the two lowest doublet excited states on TTM-TPA are 1.84 eV (D_1_) and 2.81 eV (D_2_) above the ground state. The analysis of the natural transition orbitals for fragments in Supplementary Fig. 18 shows that D_1_ of TTM-TPA is an intramolecular charge-transfer (intra-^2^CT) excitation, whereas D_2_ has a dominant locally excited (^2^LE) character on the TTM moiety. The computed excited-state energies for all of the selected molecular complexes are shown in Fig. 5a. Excitations localized on each fragment show relatively narrow energy distributions, with a slightly larger standard deviation for D_1_, as expected from its intra-^2^CT character. The calculations also suggest the presence of a much broader distribution of inter-^2^CT excitations (mostly involving transitions from the anthracene core of MADN to the TTM moiety; Supplementary Fig. 21) that are energy-resonant with D_1_ and T_1_, and could thus potentially act as mediating states in triplet–doublet energy transfer. The large energy range spanned by these inter-^2^CT states originates from the heterogeneous conformational and electrostatic landscape in amorphous solids^41,42^. These results (that is, the near degeneracy of inter-^2^CT with D_1_ and T_1_) should be taken with caution since the optimization of isolated molecular pairs might facilitate the formation of strongly interacting complexes difficult to encounter in the real system. Hence, optimized CPs are expected to exhibit shorter intermolecular distances than those in the amorphous material, triggering an overstabilization of charge-separated states. Indeed, when inter-^2^CT states are directly computed on molecular dynamics molecular pairs, transition energies are considerably higher (Supplementary Table 10).

Excitation energy transfer (EET) rates for the S_1_–D_1_ and S_1_–D_2_ processes were computed using the Marcus–Levich–Jortner equation (Methods), in which all of the key parameters (that is, reorganization energies, Huang–Rhys factors, electronic couplings and energy differences) were obtained by quantum-chemical calculations. For **CP1**, we calculate an EET time constant from S_1_ to D_1_ of 3 ps (excluding outliers, an average value of 9 ps is obtained for the investigated pairs; Supplementary Table 12). Despite the smaller energy offset between the states, the corresponding EET time constant from S_1_ to D_2_ is much longer at 20 ps (23 ps on average) due to reduced Coulomb coupling and smaller oscillator strength associated with D_2_ compared with D_1_. We conclude that singlet–doublet energy transfer occurs primarily through the S_1_–D_1_ pathway on timescales of a few picoseconds, in excellent agreement with experiment.

We now turn to triplet–doublet energy transfer. A triplet state interacting with a radical can form either an overall doublet or quartet encounter pair in a statistical ratio of 1:2 (Fig. 5b)^21^. For overall doublet pairs, triplet–doublet energy transfer can occur with spin conservation. The quantum-mechanical coupling between states can take the form of a two-electron exchange integral, as in Dexter energy transfer. However, the presence of nearby inter-^2^CT excitations also supports a superexchange-mediated mechanism, in which the effective coupling is proportional to the product of two—typically much larger—one-electron matrix elements^38^. Building on the pure spin-states of individual fragments, both the direct two-electron and the indirect one-electron electronic couplings were computed for the same pairs as above (Supplementary Section 4). Our calculations show that a direct exchange mechanism provides very slow T_1_–D_1_ energy transfer times, with values approaching tens of nanoeconds in some pairs. However, superexchange couplings are extremely sensitive to the wave function overlap and in turn to the CP geometry; thus, for some pairs, they bring the energy transfer timescales down to tens of picoseconds (which is in the same range as CT-mediated triplet–doublet energy transfer in related covalently linked radical-chromophore molecules)^38^. It is likely that the conversion from the host triplet to the emissive doublet states is limited by diffusion of the triplet excitations within the MADN host. As a first step towards the modelling of triplet diffusion, we computed T_1_ hopping rates to all nearest neighbours of three randomly selected MADN molecules (Supplementary Section 4). Although the values vary over multiple orders of magnitude, the fastest event for the three cases approaches a few tens of nanoseconds (Supplementary Table 15), which is typically orders of magnitude slower than the T_1_–D_1_ energy transfer. We thus conclude that the thermally activated delayed radical emission is controlled by triplet diffusion within the host, which limits the rate of (re)formation of overall doublet encounter pairs.

## Conclusion

Electrical excitation with a fast charge-transporting host leads to the generation of singlet and triplet exciton states that can be harvested by doublet radicals towards highly efficient NIR EL in OLEDs. Here, the handling of excitations mitigates the energy gap law for non-radiative decay by a design that combines high-energy S_1_ and low-energy T_1_ excitons of the host with matching to low-energy D_1_ excitons of the radical emitter. The principle is demonstrated using the MADN:TTM-TPA combination, which shows rapid singlet–doublet transfer (*τ* = 8 ps) upon photoexcitation and reversible doublet–triplet cycling with efficient delayed emission (*τ* > 0.16 µs). The luminescent NIR radical system is implemented in high-performing OLEDs with a maximum EQE of 9.6% for EL at 800 nm that operate to the high maximum radiance of ∼68,000 mW sr^−1^ m^−2^, with low efficiency roll-off and enhanced stability. Our design boosts performance in radical-based OLEDs and has broad implications for reducing non-radiative losses in devices beyond light-emitting applications with NIR light.

## Methods

### Sample preparation and device fabrication

TTM-TPA was dissolved in solvents at a concentration of 0.1 mg ml^–1^ to measure its PL and absorption spectra in solution. Organic films were made via a thermal evaporation process under high vacuum (~10^−7^ torr); 100 nm of CBP neat, MADN neat, and TTM-TPA 3% doped in CBP and MADN were deposited on glass substrates to measure steady-state PL, PLQE, transient PL and transient absorption. For the fabrication of OLEDs and single-carrier devices, indium-tin-oxide-coated substrates were cleaned with acetone and isopropyl alcohol, and then O_2_ plasma treatment was applied to align the energy level with a hole transporting layer. All layers, including organic layers and a LiF/aluminium cathode, were thermally deposited under high vacuum (~10^−7^ torr). The doping concentrations stated in this study denote weight percentages.

### Steady-state photophysical measurements

Steady-state PL spectra were measured using an Edinburgh Instruments fluorescence spectrometer (FLS980) with a monochromated xenon arc lamp at *λ*_ex_ = 330 nm and 370 nm for CBP and MADN, respectively, under nitrogen flow. A Shimadzu UV-3600 Plus spectrophotometer was employed to measure absorption spectra. A FLS980 with an integrating sphere under nitrogen flow was used to measure PLQY; the films were excited by 330 nm and 370 nm lasers for CBP- and MADN-based films, respectively.

### Device characterization

The *J*–*V* characteristics of single-carrier devices were recorded with a Keithley 2635 source-meter. The performance of the OLED devices was measured with a Keithley 2635 source-meter and a calibrated silicon photodiode. The EL spectra were recorded by an Ocean Optics Flame spectrometer. The device lifetime was measured using a calibrated silicon photodiode recording EL intensity with time at the constant current density of 0.1 mA cm^−1^. The transient EL characteristics are recorded using an Andor spectrometer set-up (Andor SR303i) with an electrically gated intensified charge-coupled device camera (Andor iStar DH740 CCI-010). The voltage pulse was given by a Keithley 2401 function generator (100 kHz frequency and 1 µs pulse width). For MEL measurements, an EL device was positioned between magnet cores (GMW 3470 electromagnet) and a Keithley 2635 source-meter was used to apply the voltage to the device; its EL spectrum was recorded with an Andor spectrometer (Shamrock 303i and iDus camera) with and without a magnetic field.

### Time-resolved spectroscopic measurements

Sample excitation with a laser pump pulse was provided by a frequency-doubled 800 nm pulse from titanium:sapphire amplifier (Spectra Physics Solstice Ace, 100 fs pulses at 800 nm, 7 W output at 1 kHz). Transient PL was recorded for the encapsulated films by using an Andor electrically gated intensified charge-coupled device intensified charge-coupled device with 330 nm laser excitation for CBP, 400 nm laser excitation for MADN, and 532 nm laser excitation for TTM-TPA; the decay kinetics were obtained from the integration of the total spectrum at each time. An optical cryostat (Oxford Instruments) was used to measure the temperature-dependent transient PL under high vacuum (~10^−5^ mbar).

Short-time transient absorption studies at different wavelengths of excitation were achieved from the wavelength-tuneable output of a TOPAS commercial optical parametric amplifier (Light Conversion), which was pumped by the 800 nm laser pulses from the titanium:sapphire amplifier. The pump pulses were chopped at 500 Hz to enable shot-to-shot referencing, which accounted for intensity fluctuations in the amplifier. Probe pulses for transient absorption were obtained from a set of home-built non-colinear optical parametric amplifier systems for the visible (510–790 nm) and infrared (1,250–1,650 nm) wavelength ranges. The non-colinear optical parametric amplifier probe pulses were divided into two identical beams by a 50/50 beamsplitter; this allowed for the use of a second reference beam for an improved signal:noise ratio. The probe pulses were detected by silicon (Hamamatsu S8381-1024Q) and InGaAs (Hamamatsu G11608-512DA) dual-line array with a custom-built board from Stresing Entwicklungsbüro.

### Modelling of energy transfer

Supplementary Section 4 discusses the modelling of energy transfer more in detail.

### Single molecule calculations

The host MADN and radical TTM-TPA structures were optimized at the DFT level with the ωB97X-D functional and the 6-31G(d,p) basis set (we note that unrestricted Kohn–Sham (UKS) DFT was used for the open-shell molecule). In performing geometry optimizations (both for the fragments and the molecular complexes; see below), we opted for the ωB97X-D functional thanks to its demonstrated reliability in characterizing ground-state geometries^43^. Yet we chose to use the RSH LC-ωhPBE functional to improve the accuracy in the calculations of electronic properties, especially in computing vertical excitation energies^44^. On each optimized molecular fragment, the range-separation parameter *ω* was gap-tuned when using the LC-ωhPBE/6-311 G(d,p) level of theory, following a procedure described elsewhere^45^. An optimally tuned (OT) *ω* value was found at 0.133 bohr^−1^ for MADN, and at 0.108 bohr^−1^ for radical TTM-TPA. Time-dependent DFT calculations were then performed on MADN and TTM-TPA, using the TDA^40^, resorting to an OT-SRSH approach, and by setting the macroscopic dielectric constant to that of toluene (2.37). All (TD)DFT calculations were performed using the GAUSSIAN16 suite^46^, unless stated otherwise.

### Sample preparation

Classical molecular dynamics simulations were performed to build an amorphous MADN:TTM-TPA sample; the simulation comprised 324 host MADN molecules doped with six TTM-TPA radicals at a m/m concentration of 3.1%. The general AMBER force-field^47^ for organic molecules was used, in which atomic electrostatic potentials charges were computed at the DFT ωB97X-D/6-311++G(d,p) level of theory on the previously optimized species.

### Molecular complexes calculations

Each MADN:TTM-TPA pair was selected according to geometrical criteria: a coarser selection on the molecular CP centre of mass distance, shorter than 12 Å, and a tighter one on the atom–atom distance, shorter than 4 Å. The structures of the selected complexes (36 in total) were further relaxed by means of UKS DFT ωB97X-D/6-31G(d,p). After the DFT optimization, the pair labelled **CP1** was identified as the most stable in terms of ground-state total energy. On that specific MADN:TTM-TPA pair, the *ω* value was gap-tuned at 0.095 bohr^−1^ by using the LC-ωhPBE/6-311G(d,p) level of theory. OT-SRSH UKS TDA TDDFT calculations were then performed in toluene on 17 complexes (the first ten were selected according to the energy rank, whereas the other seven were randomly chosen to scan other potential relative positions and orientations of MADN and TTM-TPA).

### Calculation of EET rates

The Marcus–Levich–Jortner equation, used to compute EET rates, reads as follows:

$$\begin{array}{l}{\kappa }_{\rm{EET}}=\frac{2\uppi }{\hslash }{V}_{\rm{EET}}^{2}\sqrt{\frac{1}{4\uppi {\lambda }_{\rm{s}}{k}_{\rm{B}}T}}\times \\ \times \sum _{n}\left\{\exp (-{S}_{\rm{eff}})\frac{{S}_{\rm{eff}}^{n}}{n!}\times \exp\left[-\frac{{(-\Delta {E}_{{\rm{S(T)}}_1-{\rm{D}}_{1(2)}}^{\,0}+{\lambda }_{\rm{s}}+n\hslash {\omega }_{\rm{eff}})}^{2}}{4{\lambda }_{\rm{s}}{k}_{\rm{B}}T}\right]\right\}\end{array}$$

where ${\Delta E}_{{\rm{S(T)}}_{{1}}-{\rm{D}}{_{1(2)}}}^{0}$ is the energy difference between two excited states (either S_1_ or T_1_ in MADN and D_1_ or D_2_ in TTM-TPA) and in this work was taken from OT-SRSH UKS TDA TDDFT calculations; *V*_EET_ is the electronic coupling associated to the EET process (see below); *λ*_s_ is the external reorganization energy; and *S*_eff_ is the Huang–Rhys factor describing the coupling of the energy transfer to an effective, internal normal mode of frequency *ω*_eff_, where *S*_eff_ was obtained directly from the internal reorganization energy *λ*_i_ as *S*_eff_ = *λ*_i_/ℏ*ω*_eff_.

### Calculation of reorganization energies

We used a displaced harmonic oscillator model to compute internal (*λ*_i_) and external (*λ*_s_) reorganization energies contributions of both the host MADN and radical TTM-TPA to the different EET processes. In this model, each intramolecular normal mode is projected on the vector describing the structural changes between the optimized ground-state and excited-state geometries, thereby partitioning the reorganization energy into mode contributions. A vibrational analysis was performed for both the optimized S_0_ ground state and S_1_ and T_1_ excited states of MADN, and also for the optimized D_0_ ground state, and D_1_ and D_2_ excited states of TTM-TPA. Ground-state geometry optimization on fragments were performed as previously described, whereas excited-state optimizations were performed at the UKS TDA TDDFT ωB97X-D/6-31G(d,p) level of theory. All of the frequencies were computed and confirmed to be positive, and the normal modes decomposition to the reorganization energy was performed using the MOMAP software^48^.

### Calculation of electronic couplings

The electronic coupling between two interacting molecules can be computed—within a first-order perturbative approximation—directly from the transition densities of the non-interacting fragments. This so-called direct coupling (DC) scheme was developed in the TDDFT framework under the assumption that the molecular orbitals can be well separated into two fragments^49^. In such a scheme, the EET coupling can be written as:

$$\begin{array}{l}{V}_{\rm{DC}}={V}_{\rm{Coul}}+{V}_{\rm{xc}}+{V}_{\rm{ovl}}=\\ =\int d{\textbf{r}}d{\textbf{r}}^{\prime} {\rho }_{\rm{D}}^{{\rm{tr}}\ast }({\textbf{r}})\frac{1}{|{\textbf{r}}-{\textbf{r}}^{\prime} |}{\rho }_{\rm{A}}^{\rm{tr}}({\textbf{r}}^{\prime} )-\int d{\textbf{r}}d{\textbf{r}}^{\prime} {\rho }_{\rm{D}}^{{\rm{tr}}\ast }({\textbf{r}}){g}_{\rm{xc}}({\textbf{r}},{\textbf{r}}^{\prime} ){\rho }_{\rm{A}}^{\rm{tr}}({\textbf{r}}^{\prime} )\\-{\omega }_{0}\int d{\textbf{r}}{\rho }_{\rm{D}}^{\rm{tr}\ast }({\textbf{r}}){\rho }_{\rm{A}}^{\rm{tr}}({\textbf{r}})\end{array}$$

where *ρ*_D_ is the transition density of the donor fragment, corresponding to the S_1_ excited state localized on the host MADN; *ρ*_A_ is the transition density related to the D_1_ (or D_2_) excited states of TTM-TPA; *ω*_0_ is the average transition energy; and *g*_xc_ is the exchange-correlation kernel given by the used functional. Here, electronic couplings were computed using OT-SRSH UKS TDA TDDFT calculations in the gas phase at the LC-ωhPBE/6-311G(d,p) level of theory. The three terms on the right-hand side represent the coulomb, the exchange-correlation and the overlap contribution to the whole coupling, respectively.

### Triplet exciton diffusion

We built three different clusters (A, B and C) composed of MADN dimers (that is, a cluster centred on a randomly selected MADN plus its first shell of solvation) in an effort to quantify the diffusion rates of molecular triplets localized on the host matrix. We then performed OT-SRSH TDA TDDFT calculations in the gas phase at the LC-ωhPBE/6-311G(d,p) level of theory and then computed the electronic couplings for triplets of all of the nearest MADN dimers by applying a multi-state diabatization procedure^50,51^. The corresponding triplet energy transfer rates were then computed by using the Marcus–Levich–Jortner equation, in which the energy difference ${\Delta E}_{\rm{T}}^{0}$ between the initial and final triplet excited states was set to zero and the reorganization energies were calculated as explained above.

### Angle-dependent PL measurements

Angle-dependent PL measurements were conducted using a rotational stage, a half-cylindrical lens and a polarizer with 400 nm laser excitation to characterize the internal propagation angle distribution of photons in the substrate. Photoluminescence spectra were recorded from 0° to 90° using an Andor spectrometer (Shamrock 303i) with an Andor iDus charge-coupled device array. We have fitted the measured angular distribution with a transfer-matrix formalism calculation by varying the ratio between the vertical and horizontal dipoles^52,53^.

### Cyclic voltammetry

The cyclic voltammetry measurements on TTM-TPA were performed using a CHI660E electrochemical analyser with a glass carbon disk as the working electrode, a platinum wire as the counter electrode, and Ag/Ag+ as the reference electrode. TTM-TPA was dissolved in dichloromethane at a concentration of 1 mM, a sweep rate of 50 mV s^−1^ was used and the ferrocenium/ferrocene redox couple was used as an internal standard.

### Photostability

The photostability experiment for TTM-TPA diluted in toluene (200 µM) was conducted under continuous-wave ultraviolet illumination in FLS980 with a monochromated xenon arc lamp at *λ*_ex_ = 400 nm, as well as pulsed (200 fs) ultraviolet illumination at an extremely high fluence (2,800 µJ cm^−2^).

### Ultraviolet photoelectron spectroscopy

For the ultraviolet photoelectron spectroscopy, 50-nm-thick TTM-TPA film was deposited on an indium tin oxide substrate in a vacuum chamber (~10^−7^ torr). The sample was loaded into a transfer vessel in a glove box and transferred to a nitrogen chamber. Ultraviolet photoelectron spectroscopy measurements were taken in a ultra-high vacuum chamber of a photoelectron spectroscopy system (Thermo Scientific ESCALAB 250Xi) and using a double-differentially pumped helium discharge lamp (hν = 21.22 eV) with a pass energy of 2 eV and a bias at −4 V.

## Online content

Any methods, additional references, Nature Portfolio reporting summaries, source data, extended data, supplementary information, acknowledgements, peer review information; details of author contributions and competing interests; and statements of data and code availability are available at 10.1038/s41566-024-01458-3.

## Supplementary information

Supplementary Information Supplementary Figs. 1–27 and Tables 1–16.

## Data Availability

The data underlying all figures in the main text are publicly available from the University of Cambridge Repository at 10.17863/CAM.107508.
