# Supplementary material for: Efficient near-infrared organic light-emitting diodes with emission from spin doublet excitons
Source: Nat Photonics. 2024 Jun 19;18(9):905–12. doi: 10.1038/s41566-024-01458-3 (PMC11374703; doi:10.1038/s41566-024-01458-3)
Supplement: Supplementary file 1 — Supplementary Figs. 1–27 and Tables 1–16. [file 41566_2024_1458_MOESM1_ESM.pdf]

nature photonics

Supplementary information https://doi.org/10.1038/s41566-024-01458-3

Efficient near-infrared organic light-emitting diodes with emission from spin doublet excitons

In the format provided by the authors and unedited

Contents

S1. Experimental

S2. Single carrier device analysis

S3. The analysis of exciton energy transfer kinetics

S4. Modelling of energy transfer

S5. Angle-dependent PL measurement

S6. Cyclic voltammetry measurement

S7. Photostability

S8. Ultraviolet photoelectron spectroscopy

S9. References

S1. Experimental

Sample preparation and device fabrication

To measure photoluminescence (PL) and absorption spectra in solution, TTM-TPA was dissolved in solvents with 0.1 mg/ml. Organic films were made by a thermal evaporation process under high vacuum (~10^{-7} torr). 100 nm of CBP and MADN neat and TTM-TPA 3% doped in CBP and MADN were deposited on glass substrates to measure steady-state PL, PLQE, transient PL, and transient absorption. For the fabrication of OLEDs and single-carrier devices, ITO-coated substrates were cleaned with acetone and isopropyl alcohol, and then O_2 plasma treatment was applied to align the energy level with a hole transporting layer. All layers, including organic layers and a LiF/aluminium cathode, were thermally deposited under high vacuum (~10^{-7} torr). The doping concentrations stated in this study denote weight percentages.

Steady-state photophysical measurements

Steady-state PL spectra were measured by an Edinburgh Instruments fluorescence spectrometer (FLS980) with a monochromated xenon arc lamp at λ_{Ex} = 330 nm for CBP and λ_{Ex} = 370 nm for MADN under a nitrogen flow. Shimadzu UV-3600 Plus spectrophotometer was employed for the measurement of absorption spectra. FLS980 with an integrating sphere under a nitrogen flow was used to measure PL quantum yield (PLQY), and the films were excited by 330 nm laser for CBP-based films and 400 nm laser for MADN-based films.

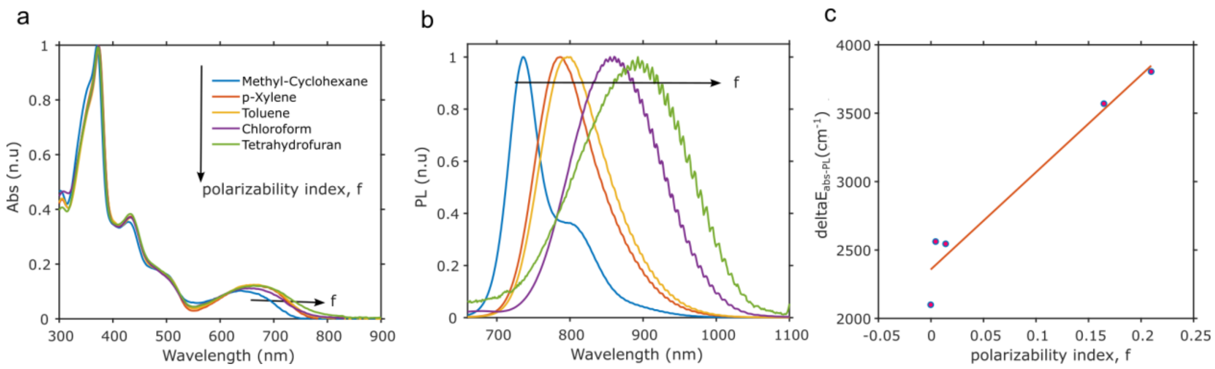

Supplementary Fig. 1 | PL and absorption spectra of TTM-TPA in different solvents. **a,** Steady-state ultraviolet–visible and **b,** photoluminescence spectra of TTM-TPA (0.1 mg/ml) in various solvents with various polarizability index, f. Photo excitation wavelength is 532 nm. **c,** Lippert–Mataga plot of the Stokes shift (ν_a–ν_f) versus f for TTM-TPA (circles). ν_a and ν_f denote the absorption and fluorescence energies, respectively.

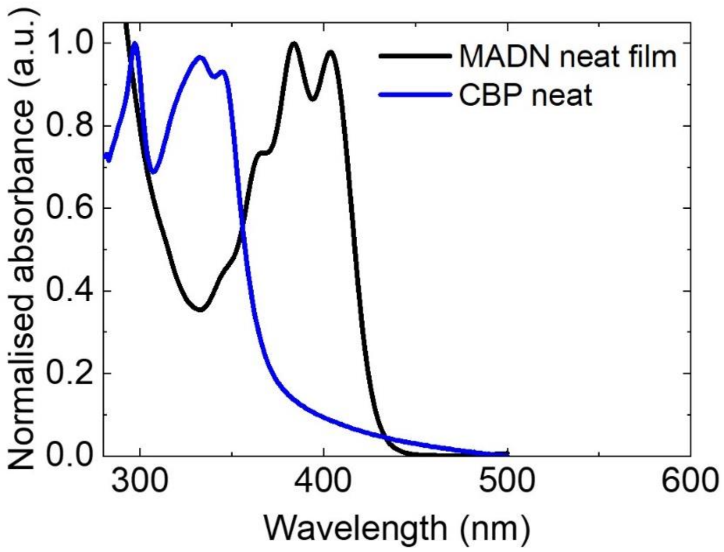

Supplementary Fig. 2 | The normalised absorbance of CBP and MADN neat film (100 nm).

Device characterisation

The J-V characteristics of single-carrier devices were recorded by a Keithley 2635 source-meter. The performance of the OLED devices was measured by a Keithley 2635 source-meter and a calibrated Si photodiode. The EL spectra were recorded by an Ocean Optics Flame spectrometer. The device lifetime was measured by a calibrated Si photodiode recording EL intensity with time at the constant current density of 0.1 mA cm^{-1}. The transient EL

characteristics are recorded by an Andor spectrometer setup (Andor SR303i) with an electrically gated ICCD camera (AndoriStar DH740 CCI-010). The voltage pulse was given by a Keithley 2401 function generator (100 kHz frequency and 1μs pulse width). For MEL measurements, an EL device was positioned between magnet cores (GMW 3470 electromagnet) and a Keithley 2635 sourcemeter was utilised to apply the voltage to the device, and its EL spectrum was recorded by an Andor spectrometer (Shamrock 303i and iDus camera) with and without a magnetic field.

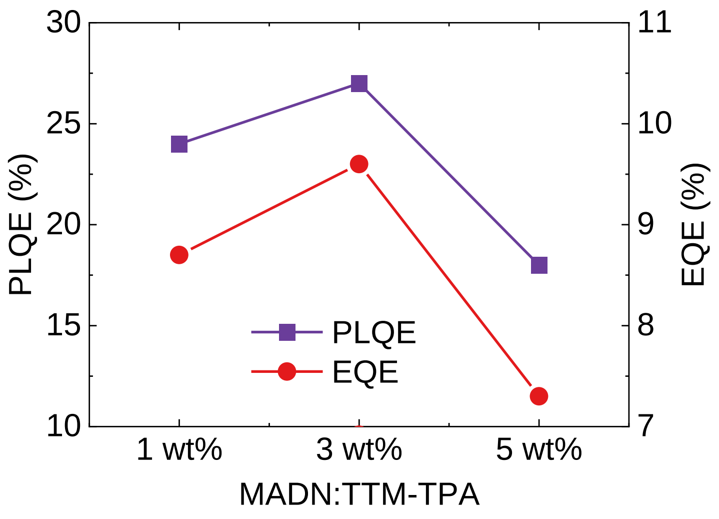

Supplementary Fig. 3 | Concentration dependence for PLQE and EQE of MADN:TTM-TPA films and devices.

Supplementary Table 1 | Summary of device performance CBP:B3PYMPM:TTM-TPA 3% device.

	V_{on}[a)] (V)	EQE_{Max} (%)	$EQE_{J-1.0}$[b)] (%)	$EQE_{J-100.0}$[c)] (%)	$Radiance_{Max}$ (mW sr^{-1} m^{-2})	λ_{max} (nm)
CBP:B3:TTM-TPA 3%	3.2	5.5	4.4	3.1	48,000	820

[a)]Voltage at 10^{-1} mW sr^{-1} m^{-2}, [b)]EQE at 1 mA cm^{-2}, [c)]EQE at 100 mA cm^{-2}.

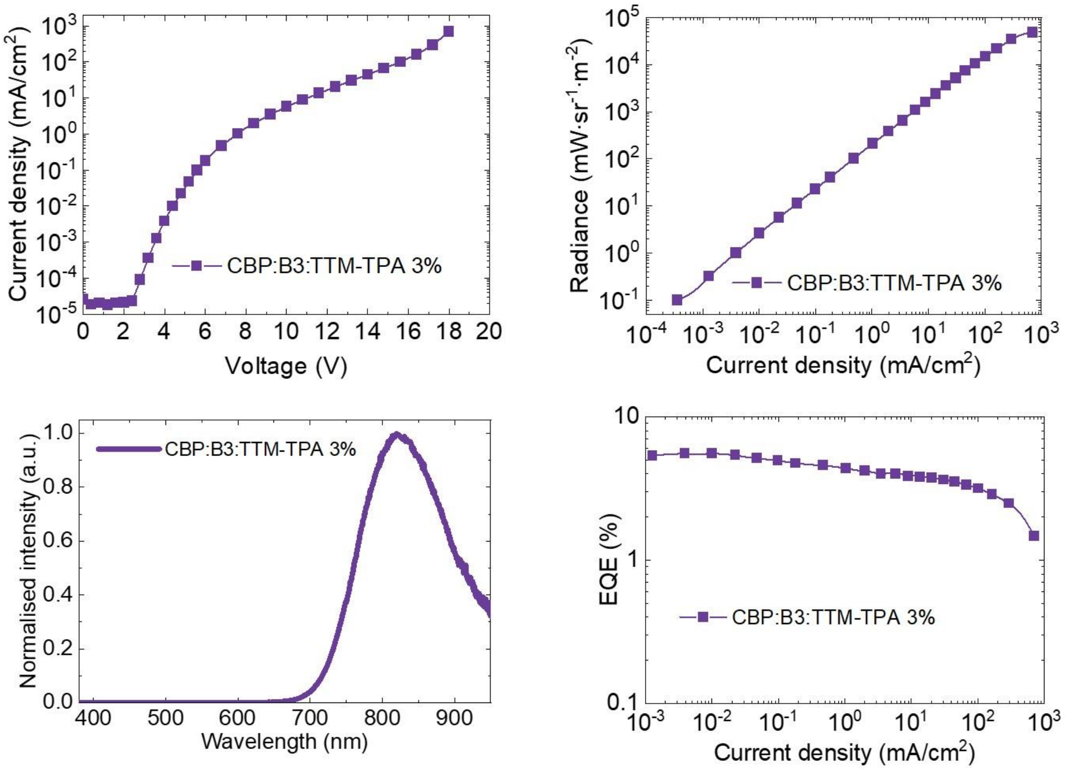

Supplementary Fig. 4 | Optoelectronic performance for CBP:B3PYMPM:TTM-TPA 3% device.

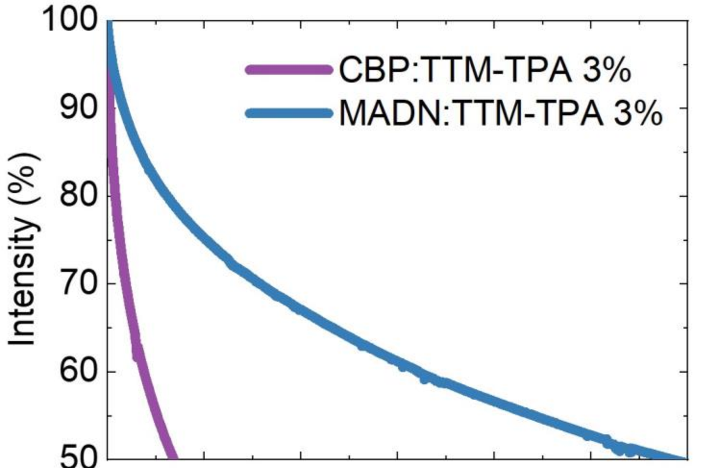

Supplementary Fig. 5 | Lifetime for the devices measured at 0.1 mA cm^{-2}.

Supplementary Table 2 | Summary of NIR OLED device performance (between 780 nm and 900 nm) from published literature.

Emission Concept	Materials	Peak wavelength (nm)	$Radiance_{Max}$ ($mW\ sr^{-1}\ m^{-2}$)	EQE_{Max} (%)	Reference
Fluorescence	TBTTT	880	400	0.13	[6][1]
	BTT	840	4,800	1.09	[7][2]
Phosphorescence	Pt-TPTNP	900	5,400	3.8	[8][3]
	Ir(dtbpa)3	811	300	0.5	[9][4]
	Pt(tptnp)	886	5,700	3.8	[10][5]
	4Me	890	46,400	2.3	[11][6]
TADF	BF2 derivative	782	-	0.27	[12][7]
Sensitized fluorescence	BPPC	790	-	5.4	[13][8]
	BPPC-Ph	841	-	3.5	[14][9]
	NZ2mDPA	786	4,600	0.77	[15][10]
Doublet fluorescence	TPA-PyBTM’	805	600	6.4	[16][11]
	TPA-PyBTM’	820	4,000	4.7	
	TPA-PyBTM’	820	3,000	3.7	
	**TTM-TPA**	**820**	**8,100**	**6.1**	**This work**
	**TTM-TPA**	**800**	**68,000**	**9.6**	**This work**

Supplementary Table 3 | Summary of device performance for MADN:TTM-TPA devices with 1 and 5% doping.

	V_{on}[a)] (V)	EQE_{Max} (%)	$EQE_{J-1.0}$[b)] (%)	$EQE_{J-100.0}$[c)] (%)	$Radiance_{Max}$ (mW sr^{-1} m^{-2})	λ_{max} (nm)
MADN:TTM-TPA 1%	2.3	8.7	5.6	3.0	41,300	780
MADN:TTM-TPA 5%	2.4	7.3	4.3	2.5	39,600	800

[a)]Voltage at 10^{-1} mW sr^{-1} m^{-2}, [b)]EQE at 1 mA cm^{-2}, [c)]EQE at 100 mA cm^{-2}.

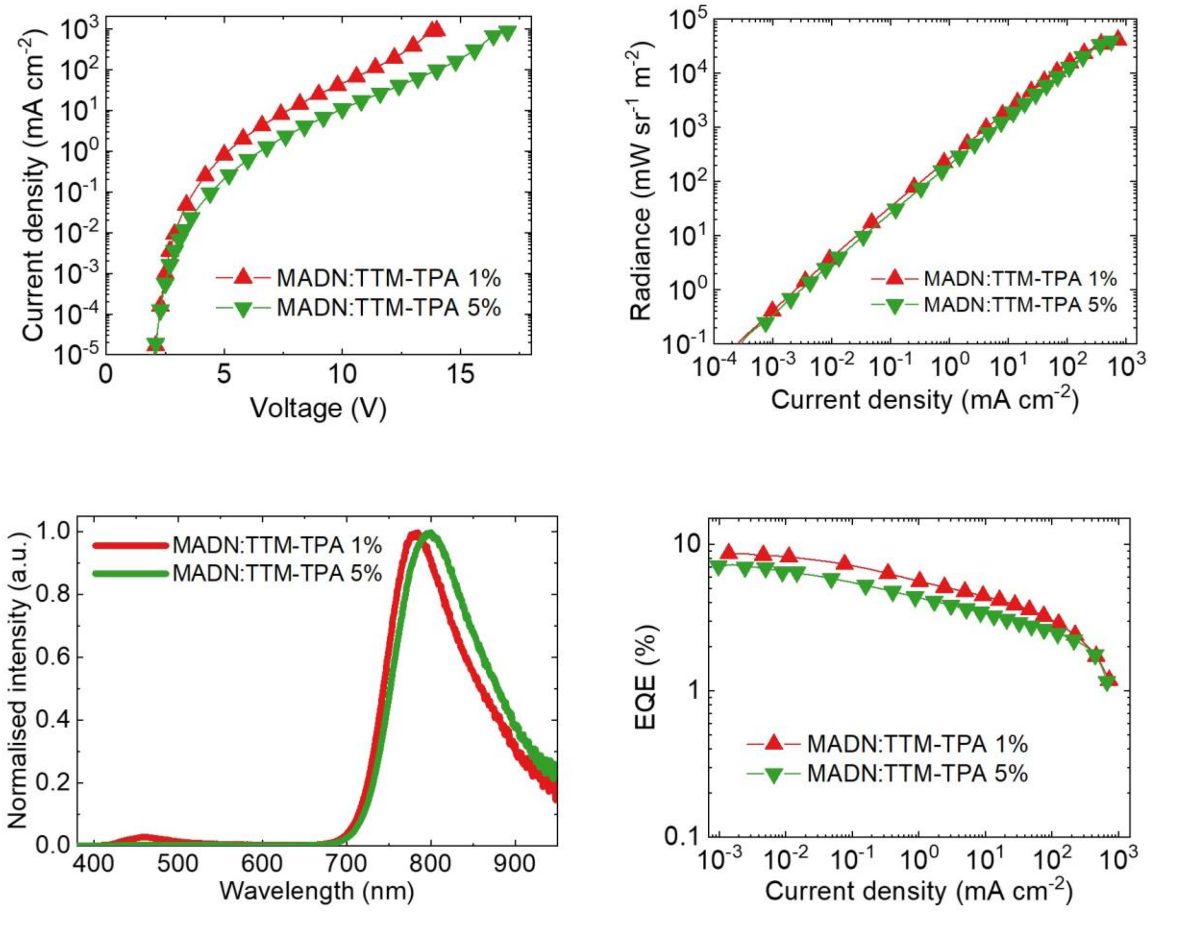

Supplementary Fig. 6 | Optoelectronic performance for MADN:TTM-TPA 1 and 5% devices.

Supplementary Table 4 | Summary of device performance for TTM-TPA-based OLEDs for different batches. The average EQE and standard deviations are 6.0% and 0.1% for the CBP:TTM-TPA device and 9.3% and 0.3% for the MADN:TTM-TPA device.

	V_{on}[a)] (V)	EQE_{Max} (%)	$EQE_{J-1.0}$[b)] (%)	$EQE_{J-100.0}$[c)] (%)	$Radiance_{Max}$ ($mW\ sr^{-1}\ m^{-2}$)	λ_{max} (nm)
CBP:TTM-TPA 3% -1	2.8	6.1	4.9	1.3	8,100	820
CBP:TTM-TPA 3% -2	2.9	5.9	4.9	1.2	7,800	820
CBP:TTM-TPA 3% -3	2.9	5.7	4.9	1.2	7,600	820
CBP:TTM-TPA 3% -4	2.8	5.9	4.5	1.1	5,700	820
CBP:TTM-TPA 3% -5	2.8	6.1	4.8	1.2	6,600	820
CBP:TTM-TPA 3% -6	2.9	6.0	4.8	1.2	6,500	820
MADN:TTM-TPA 3% -1	2.4	9.6	7.2	4.2	68,000	800
MADN:TTM-TPA 3% -2	2.4	9.1	6.6	3.4	52,000	800
MADN:TTM-TPA 3% -3	2.4	8.9	6.5	3.4	58,000	800
MADN:TTM-TPA 3% -4	2.3	9.6	7.2	4.1	65,000	800
MADN:TTM-TPA 3% -5	2.4	9.1	6.8	3.8	60,000	800
MADN:TTM-TPA 3% -6	2.4	9.4	7.3	4.2	67,000	800

[a)]Voltage at 10^{-1} mW sr^{-1} m^{-2}, [b)]EQE at 1 mA cm^{-2}, [c)]EQE at 100 mA cm^{-2}.

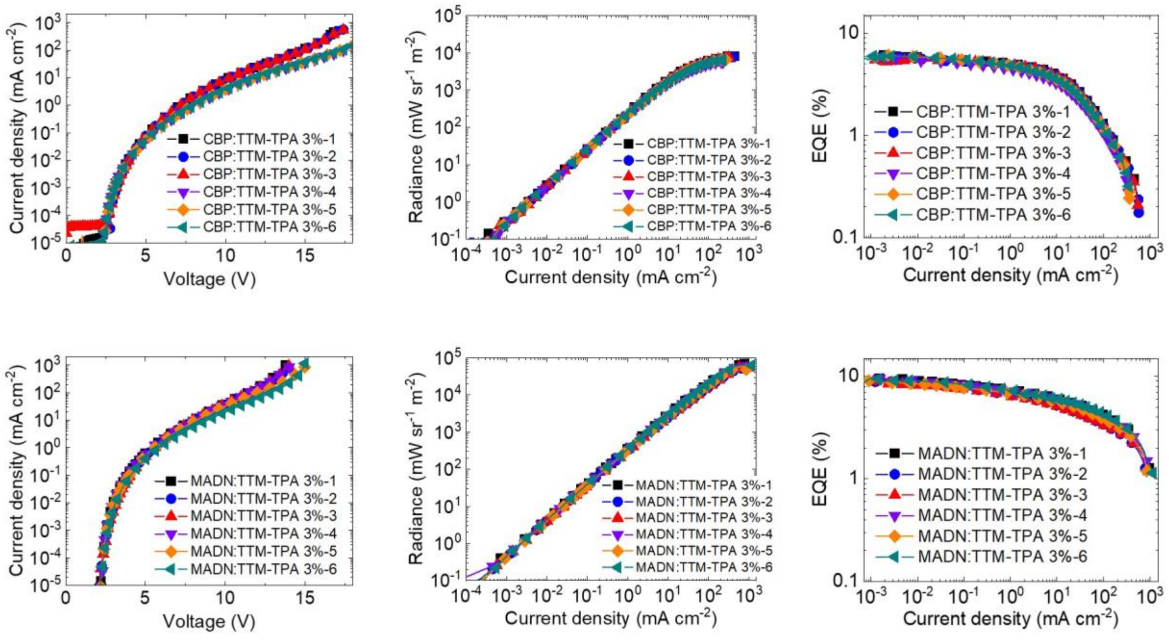

Supplementary Fig. 7 | Device reproducibility for TTM-TPA-based OLEDs.

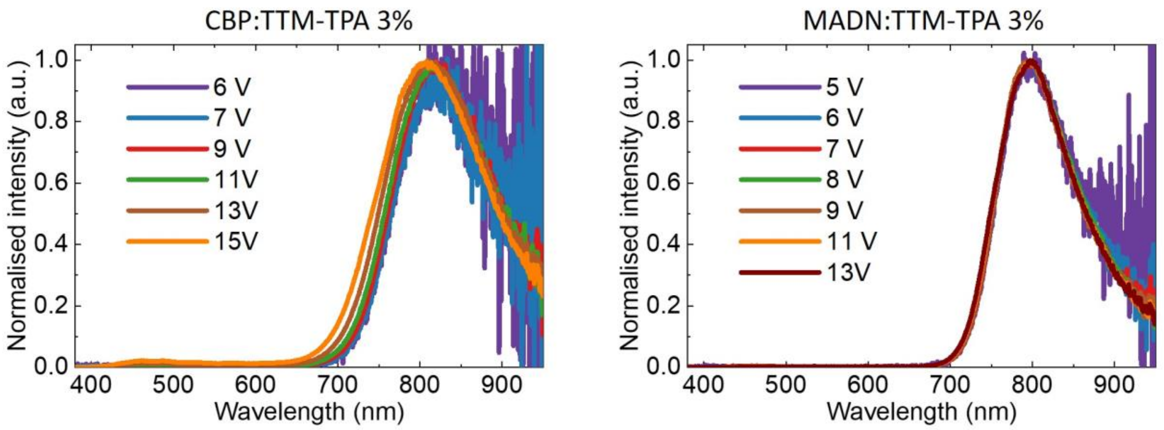

Supplementary Fig. 8 | Voltage-dependent EL spectra. Due to the poor electron mobility of CBP, the EL spectra of the CBP device are blue shifted due to emission tending to come from the host/electron transport layer interface when the emission zone is narrow. In contrast, the EL spectra of the MADN device are stable for all voltage ranges due to more balanced electrical properties and the energy transfer-based emission mechanism.

Time-resolved spectroscopic measurements

Sample excitation with a laser pump pulse was provided by a frequency-doubled 800 nm pulse from Ti:sapphire amplifier (Spectra Physics Solstice Ace, 100 fs pulses at 800 nm, 7 W output at 1 kHz). Transient PL was recorded for the encapsulated films by using an Andor electrically gated intensified charge-coupled device (ICCD) with 330 nm laser excitation for CBP, 400 nm laser excitation for MADN, and 532 nm laser excitation for TTM-TPA; the decay kinetics were obtained from the integration of the total spectrum at each time. An optical cryostat (Oxford Instruments) was utilized to measure the temperature-dependent transient PL under high vacuum (~10^{-5} mbar).

Short-time TA studies at different wavelengths of excitation were achieved from the wavelength tuneable output of TOPAS commercial optical parametric amplifier (Light Conversion), which was pumped by the 800 nm laser pulses from the Ti:sapphire amplifier. The pump pulses were chopped at 500 Hz to enable shot-to-shot referencing, which accounted for intensity fluctuations in the amplifier. Probe pulses for TA were obtained from a set of home-built non-colinear optical parametric amplifier (NOPA) systems for the visible (510-790 nm) and infrared (1250-1650 nm) wavelength ranges. The NOPA probe pulses were divided

into two identical beams by a 50/50 beamsplitter; this allowed for the use of a second reference beam for improved signal:noise. The probe pulses were detected by Si (Hamamatsu S8381-1024Q) and InGaAs (Hamamatsu G11608-512DA) dual-line array with a custom-built board from Stresing Entwicklungsbüro.

S2. Single carrier device analysis

To understand the driving force of the significantly enhanced device performance in detail, the doublet EL devices are specifically investigated based on the examination and comparison of the charge transporting properties through the studies of single-carrier devices. The hole-only device (HOD) and electron-only device (EOD) were designed based on the full device architecture to directly apply their characteristics to the full device performance, as shown in Supplementary Fig. 9. Supplementary Fig. 10 presents the comparison of the J-V plots for HODs and EODs, respectively. Regarding HODs, hole transport in MADN is more rapid than in CBP, considering the steeper J-V curves. Also, holes are less trapped at radicals in MADN than CBP due to the shallower highest occupied molecular orbital (HOMO) energy level of MADN. Also, electron transport in MADN is much faster than in CBP, leading to a more balanced population of charge carriers in the EML. Although electrons are captured at radicals in both MADN and CBP, much more enhanced electron transport in MADN is apparent compared to CBP. Therefore, MADN is capable of providing much more improved charge transport with better charge balance in the EML, leading to substantially enhanced device performance. Also, as holes are less captured in a MADN matrix, it is expected that direct recombination of hole and electron at radical sites would be much less dominant, whereas

exciton energy transfer from MADN to TTM-TPA would dominate the overall emission process in this combination.

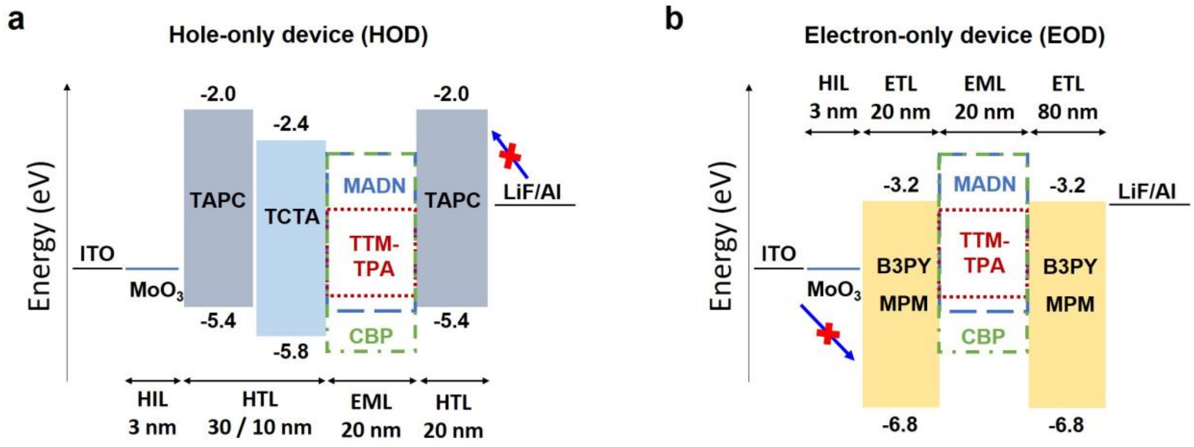

Supplementary Fig. 9 | The device architectures of HOD and EOD. a, TAPC 20 nm is deposited between the EML and cathode to preclude electron injection. **b,** EML is sandwiched by B3PYMMPM to ensure electron-only currents.

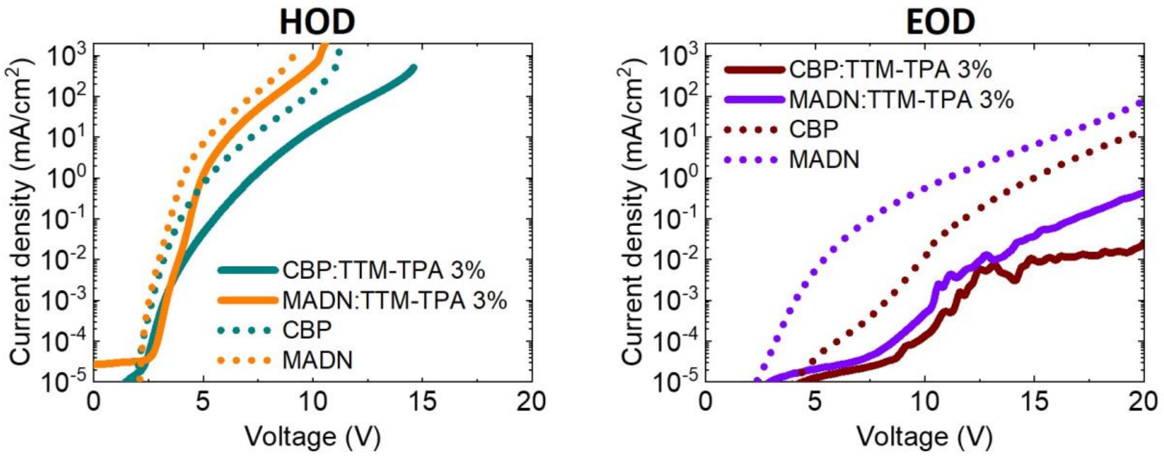

Supplementary Fig. 10 | HOD and EOD characteristics for the devices with and without TTM-TPA doping. Both hole and electron transporting properties are much higher in MADN than CBP, in line with the J-V profiles for the full devices.

S3. The analysis of exciton energy transfer kinetics

Förster resonance energy transfer (FRET)

Supplementary Fig. 11 shows the schematic diagram of exciton decay kinetics and energy transfer pathways in the MADN:TTM-TPA system. Assuming that the internal conversion (IC) time is negligibly short, and doublet excitons experiencing radiative and nonradiative decay ($k_{\mathrm{r,D}}$ and $k_{\mathrm{nr,D}}$) are generated by the FRET of singlet excitons formed by photoexcitation when MADN is excited by 400 nm, the FRET efficiency (E_{FRET}) can be extracted as,[12]

$$E_{\mathrm{FRET}} = \frac{k_{\mathrm{FRET}}}{k_{\mathrm{r,MADN}}+k_{\mathrm{nr,MADN}}+k_{\mathrm{FRET}}}$$

$$= \frac{1/\Phi_F \int P_{\mathrm{TTM-TPA}}(\lambda)d\lambda}{1/\Phi_{\mathrm{MADN}} \int P_{\mathrm{MADN}}(\lambda)d\lambda + 1/\Phi_F \int P_{\mathrm{TTM-TPA}}(\lambda)d\lambda} \qquad \text{(S1)}$$

where $k_{\mathrm{r,MADN}}$ and $k_{\mathrm{nr,MADN}}$ are the radiative and nonradiative decay rate of MADN (Supplementary Fig. 14 and Supplementary Table 5), k_{FRET} is the rate constant of the characteristic FRET, Φ_{MADN} and Φ_F are the PLQEs of the MADN and MADN-based blend. P_{MADN} and $P_{\mathrm{TTM\text{-}TPA}}$ are the PL spectra of MADN and TTM-TPA emission components, respectively, obtained from the decomposed donor and acceptor spectra from the total spectra of the MADN hosted film. From Equation S1, k_{FRET} is calculated by

$$k_{\mathrm{FRET}} = \frac{E_{\mathrm{FRET}}(k_{\mathrm{r,MADN}}+k_{\mathrm{nr,MADN}})}{1-E_{\mathrm{FRET}}} \qquad \text{(S2)}$$

Hence, from Equation S2, k_{FRET} between MADN and TTA-TPA can be extracted as 21.97×10^9/s, in line with the rapid doublet signal rise (~8 ps) of the transient absorption profile. The decay lifetime, decay rate, FRET rate, and FRET efficiency for the CBP-based and MADN-based blends are summarised in Supplementary Table 6.

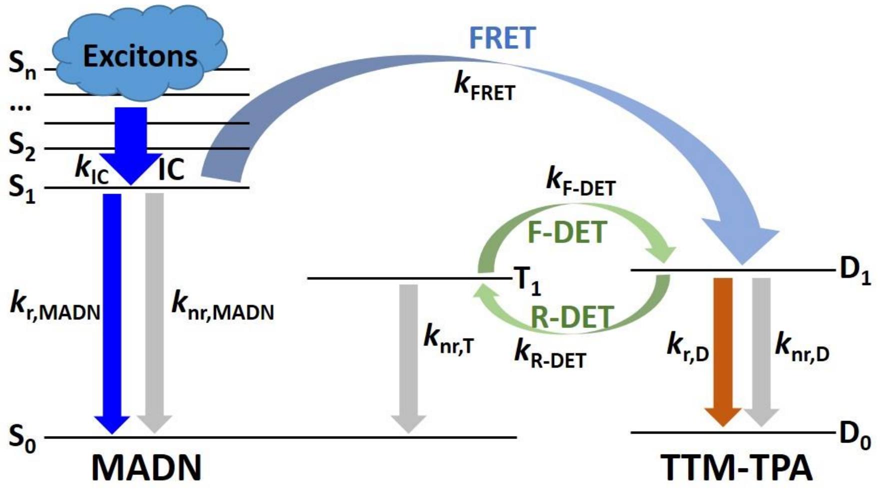

Supplementary Fig. 11 | Schematic illustration of exciton decay kinetics and intersystem energy transfer pathways between MADN and TTM-TPA.

Supplementary Table 5 | Summary of PLQE, decay lifetime, and decay rate for CBP and MADN.

	PLQE (± 0.03)	τ (10^{-9}s)[a)]	k_r (10^9/s)	k_{nr} (10^9/s)
CBP	0.78	3.27	23.85	6.73
MADN	0.41	2.23	18.39	26.56

[a)]Derived from the time when the integrated PL intensity reaches 1-(1/e).

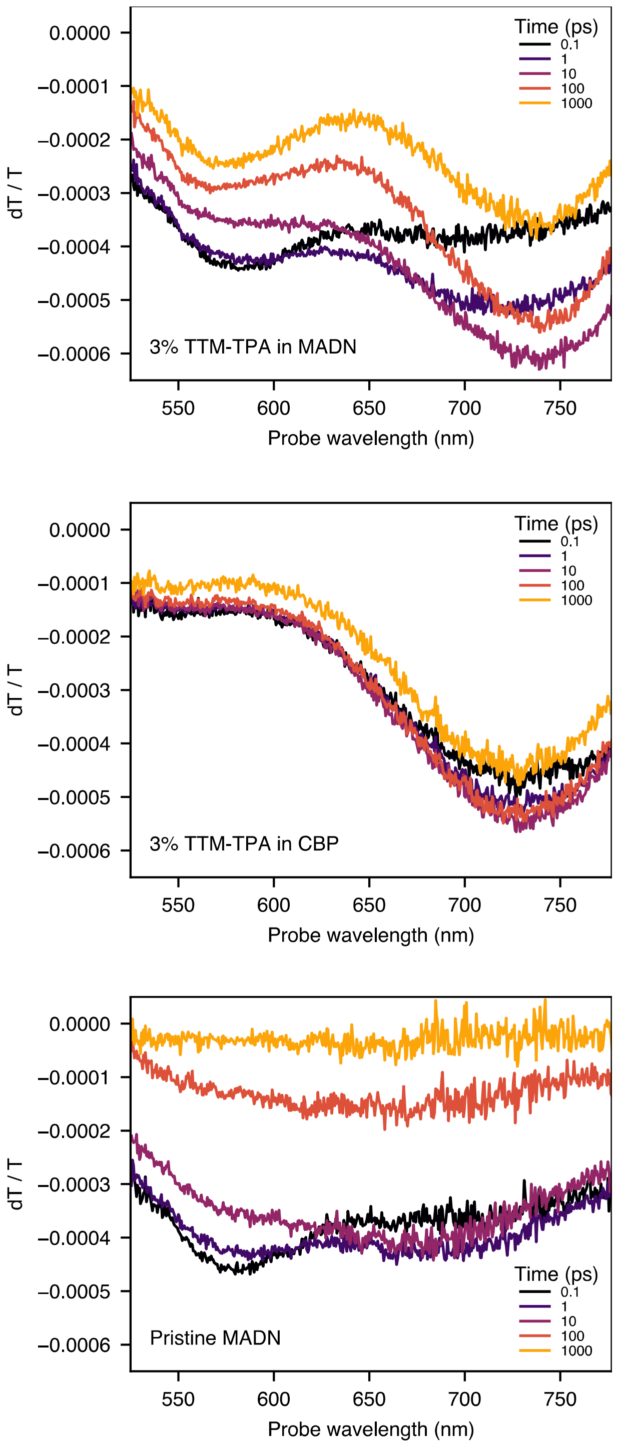

Supplementary Fig. 12 | Picosecond transient absorption spectra on encapsulated films following excitation at 400 nm pulses. Fluences are 6.4 μJ/cm^2 for 3% TTM-TPA in MADN and Pristine MADN, and 76 μJ/cm^2 for 3% TTM-TPA in CBP.

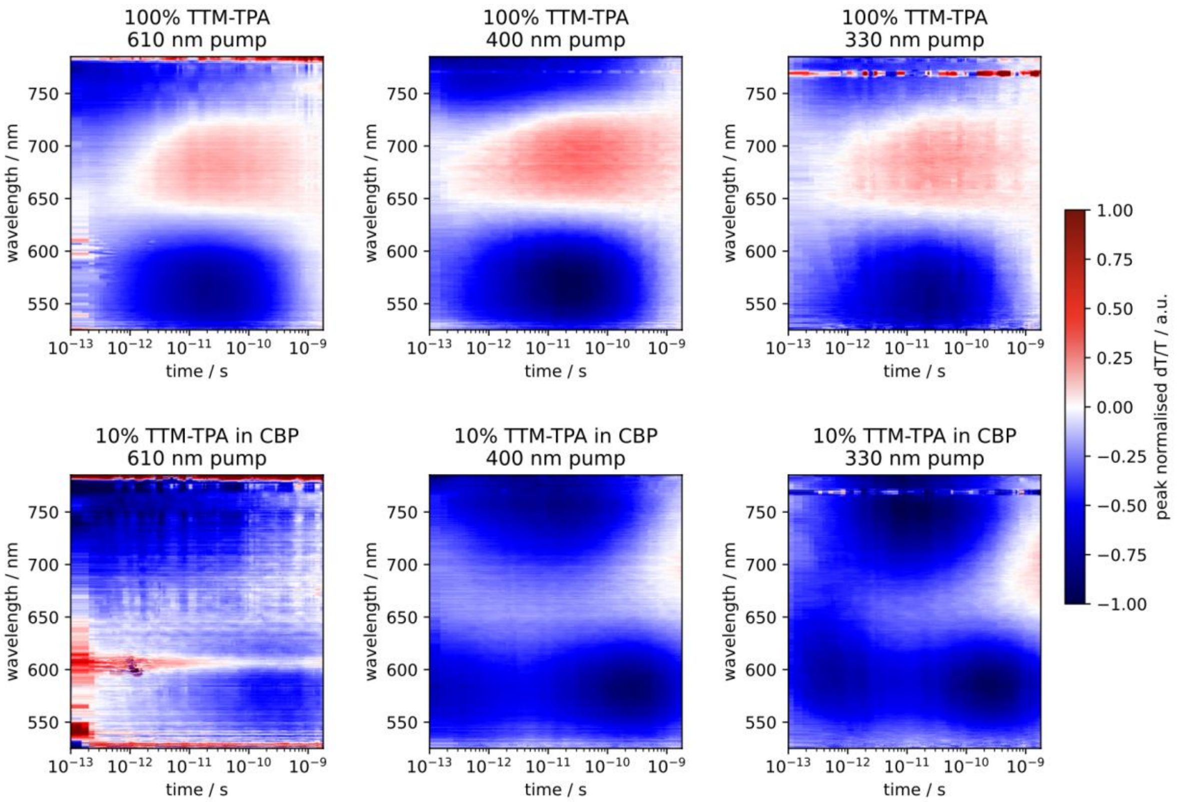

Supplementary Fig. 13 | TA on pristine TTM-TPA and its CBP films. Normalised heatmaps following excitation with 610 nm (40 μJ cm^{-2}), 400 nm (11 μJ cm^{-2}) and 330 nm (3.4 μJ cm^{-2}) pulses. Pristine TTM-TPA dynamics are broadly independent of pump wavelength, indicating D2 states prepared at shorter wavelengths rapidly internally convert to D1 states, but show non-radiative decay with lifetime near 1 ns. In the CBP blend, singlet-doublet FRET is seen when exciting above the CBP bandgap, from the CBP PIA near 600 nm to the TTM-TPA PIA near 750 nm. This occurs with lifetime of 2.7 ± 0.3 ps in the 10% blend.

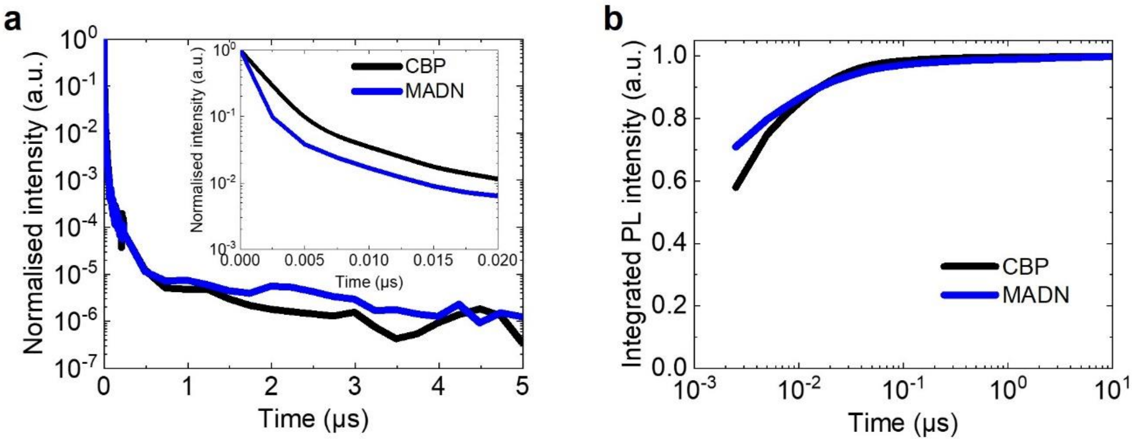

Supplementary Fig. 14 | The transient PL and normalised integrated PL intensity profiles of the CBP and MADN neat films.

Supplementary Table 6 | Summary of PLQE, decay lifetime, decay rate, FRET rate, and FRET efficiency for CBP:TTM-TPA 3% and MADN:TTM-TPA 3% photoexcited at 330 nm for CBP blend and 400 nm for MADN blend.

Host	Dopant	PLQE (± 0.02)	τ [a)] (10^{-9}s)	k_r (10^9/s)	k_{nr} (10^9/s)	k_{FRET} (10^9/s)	E_{FRET} (%)
CBP	TTM-TPA	0.19	8.27	2.30	9.79	20.08	98.5
MADN		0.27	14.33	1.88	5.09	21.97	98.0

[a)]Derived from the time when the integrated PL intensity reaches 1-(1/e).

Dexter energy transfer (DET)

To understand the mutual DET between MADN T_1 and TTM-TPA D_1, the transient PL profile of the MADN:TTM-TPA blend by the direct excitation of TTM-TPA at 532 nm was investigated (Supplementary Fig. 17). The decay profile can be separated by prompt and delayed components resulting from the intermolecular intersystem triplet-doublet DET. Accordingly, under the direct excitation of TTM-TPA in the MADN hosted blend, the doublet and triplet exciton densities in TTM-TPA and MADN (D and T) are described by,[13–15]

$$\frac{d\mathrm{D}}{dt} = -(k_{\mathrm{r,D}} + k_{\mathrm{nr,D}})\mathrm{D} - k_{\mathrm{R-DET}}\mathrm{D} + k_{\mathrm{F-DET}}\mathrm{T} \quad (S3)$$

$$\frac{d\mathrm{T}}{dt} = -(k_{\mathrm{nr,T}})\mathrm{T} - k_{\mathrm{F-DET}}\mathrm{D} + k_{\mathrm{R-DET}}\mathrm{T} \quad (S4)$$

where $k_{\mathrm{r,D}}$, and $k_{\mathrm{nr,D}}$ are the radiative and nonradiative decay rates of the doublet, $k_{\mathrm{nr,T}}$ are the nonradiative decay rate of the triplet, $k_{\mathrm{R-DET}}$ and $k_{\mathrm{F-DET}}$ are the rate constants of R-DET and F-DET. The solutions to S3 and S4 can be expressed as the simple form of the biexponential decay,

$$\mathrm{D,T} = A_1 \exp\,(-k_{\mathrm{p}}\mathrm{t}) + A_2 \exp\,(-k_{\mathrm{d}}\mathrm{t}) \quad (S5)$$

where A_1 and A_2 are the fitting parameters, and k_p and k_d are the rate constants of the prompt and delayed emission. With the intermolecular mutual DET, k_p and k_d are given by,

$$k_\mathrm{p}, k_\mathrm{d} = \frac{k_\mathrm{r,D}+k_\mathrm{nr,D}+k_\mathrm{R-DET}+k_\mathrm{nr,T}+k_\mathrm{F-DET}}{2} \times$$

$$\left(1 \pm \sqrt{1 - \frac{4(k_\mathrm{r,D}+k_\mathrm{nr,D}+k_\mathrm{R-DET})(k_\mathrm{nr,T}+k_\mathrm{F-DET})-4k_\mathrm{R-DET}k_\mathrm{F-DET}}{(k_\mathrm{r,D}+k_\mathrm{nr,D}+k_\mathrm{R-DET}+k_\mathrm{nr,T}+k_\mathrm{F-DET})^2}}\right) \quad \text{(S6)}$$

Assuming $k_\mathrm{r,D}$, $k_\mathrm{nr,D}$, and $k_\mathrm{R-DET} \gg k_\mathrm{nr,T}$ and $k_\mathrm{F-DET}$, k_p and k_d are expressed by,

$$k_p = k_\mathrm{r,D} + k_\mathrm{nr,D} + k_\mathrm{R-DET} \quad \text{(S7)}$$

$$k_\mathrm{d} = k_\mathrm{nr,T} + \left(1 - \frac{k_\mathrm{r,D}}{k_\mathrm{r,D}+k_\mathrm{nr,D}+k_\mathrm{R-DET}}\right) k_\mathrm{F-DET} = \frac{k_\mathrm{p}k_\mathrm{F-DET}-k_\mathrm{R-DET}k_\mathrm{F-DET}}{k_\mathrm{p}} \quad \text{(S8)}$$

Also, the PLQEs of the prompt and delayed emission (Φ_P and Φ_d) are given by Equation S9 and S10,

$$\Phi_\mathrm{P} = \frac{k_\mathrm{r,D}}{k_\mathrm{r,D}+k_\mathrm{nr,D}+k_\mathrm{R-DET}} \quad \text{(S9)}$$

$$\Phi_\mathrm{d} = \sum_{k=1}^{\infty}(\Phi_\mathrm{R-DET}\Phi_\mathrm{F-DET})^k\Phi_\mathrm{p} = \frac{\Phi_\mathrm{R-DET}\Phi_\mathrm{F-DET}}{1-\Phi_\mathrm{R-DET}\Phi_\mathrm{F-DET}}\Phi_\mathrm{p,D} \quad \text{(S10)}$$

where $\Phi_\mathrm{R-DET}$ and $\Phi_\mathrm{F-DET}$ are the efficiency of R-DET and F-DET, given by

$$\Phi_\mathrm{R-DET} = \frac{k_\mathrm{R-DET}}{k_\mathrm{r,D}+k_\mathrm{nr,D}+k_\mathrm{R-DET}} = \frac{k_\mathrm{R-DET}}{k_\mathrm{p}} \quad \text{(S11)}$$

$$\Phi_\mathrm{F-DET} = \frac{k_\mathrm{F-DET}}{k_\mathrm{F-DET}+k_\mathrm{nr,T}} \approx 1 \quad \text{(S12)}$$

Meanwhile, Φ_p and Φ_d are extracted experimentally from the total PLQE (Φ_total) and the intensity ratio of the prompt and delayed emission (r_1 and r_2) in the transient PL profile. Hence, Φ_total, Φ_p, and Φ_d are described by

$$\Phi_\mathrm{total} = \Phi_\mathrm{p} + \Phi_\mathrm{d} \quad \text{(S13)}$$

$$\Phi_{\rm p} = r_1 \Phi_{\rm total} \tag{S14}$$

$$\Phi_{\rm d} = r_2 \Phi_{\rm total} \tag{S15}$$

where r_1 and r_2 are expressed by

$$r_1 = \frac{A_1 \tau_{\rm p}}{A_1 \tau_{\rm p} + A_2 \tau_{\rm d}} \tag{S16}$$

$$r_2 = \frac{A_2 \tau_{\rm p}}{A_1 \tau_{\rm p} + A_2 \tau_{\rm d}} \tag{S17}$$

where A_1 and A_2 are the fitting parameters of the biexponential decay ($A_1 e^{-t/\tau_{\rm p}} + A_2 e^{-t/\tau_{\rm d}}$) for the transient PL curve, and $\tau_{\rm p}$ and $\tau_{\rm d}$ are the decay lifetime of prompt and delayed emission (Supplementary Fig. 17). Thus, from the values of $\Phi_{\rm p}$, $\Phi_{\rm d}$, $\tau_{\rm p}$, and $\tau_{\rm d}$the decay rates of the prompt and delayed emission ($k_{\rm p}$ and $k_{\rm d}$) can be extracted by the following equations,

$$k_{\rm p} = \frac{\Phi_{\rm p}}{\tau_{\rm p}} \tag{S18}$$

$$k_{\rm d} = \frac{\Phi_{\rm d}}{\tau_{\rm d}} \tag{S19}$$

From Equation S7~S12, the rate constants of R-DET and F-DET ($k_{\rm F-DET}$) are obtained by

$$k_{\rm R-DET} = \frac{\Phi_{\rm d}}{\Phi_{\rm p} + \Phi_{\rm d}} k_{\rm p} \tag{S20}$$

$$k_{\rm F-DET} = \frac{k_{\rm d} \Phi_{\rm F-DET}}{1 - \Phi_{\rm R-DET} \Phi_{\rm F-DET}} = \frac{k_{\rm p} k_{\rm d}}{k_{\rm R-DET}} \frac{\Phi_{\rm d}}{\Phi_{\rm p}} \tag{S21}$$

Therefore, $k_{\rm R-DET}$ and $k_{\rm F-DET}$ are can be obtained from S20 and S21 as 7.94 × 10^6 /s and 8.74 × 10^5 /s, respectively.

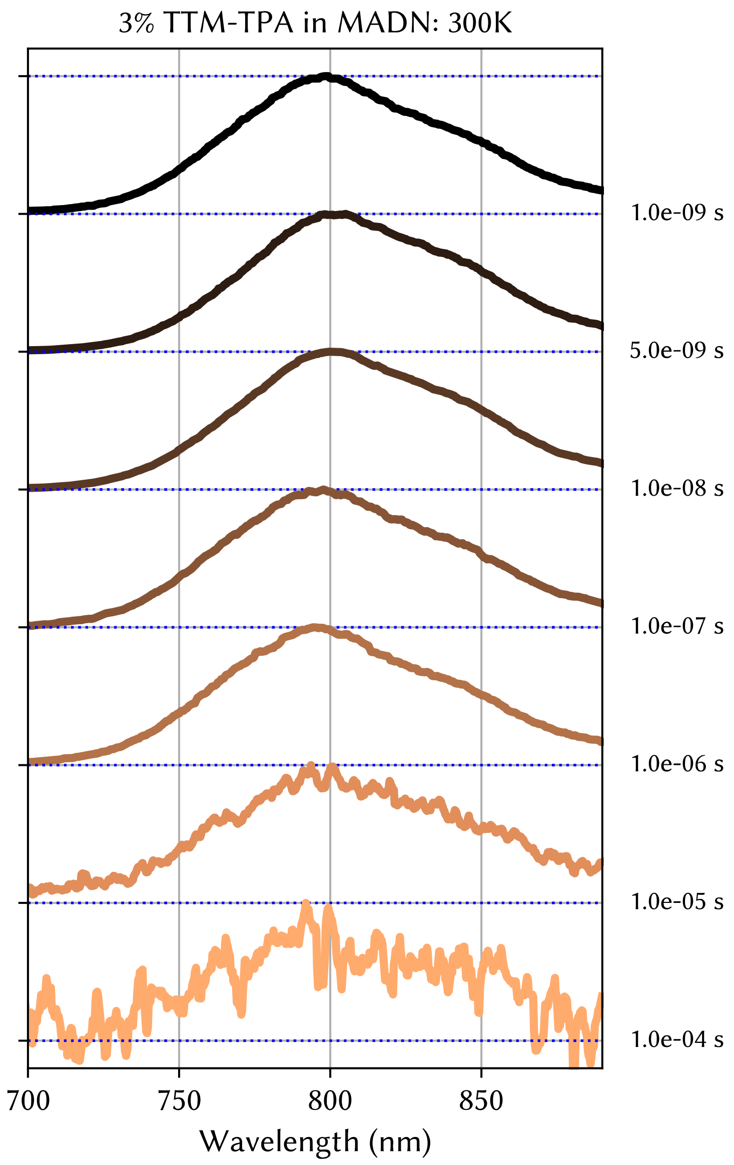

Supplementary Fig. 15 | Time-gated photoluminescence spectra of 3% TTM-TPA in MADN at temperature of 300 K following excitation at wavelength of 532 nm and fluence of 34 μJ cm^{-2}. The emission spectrum is unchanged throughout the decay across the 1 ns - 100 μs time range.

Arrhenius analysis

The activation energy (E_a) for the MADN blend can be extracted by the Arrhenius equation,

$$k = Ae^{\frac{-E_a}{k_\mathrm{B}T}}, \tag{S22}$$

where k is the rate constant, E_a is the activation energy, k_B is the Boltzmann constant (8.617 × 10^{-5} eV/K), and T is the absolute temperature. The delayed decay rates ($1/\tau_\mathrm{d}$) for the MADN:TTM-TPA blend (Supplementary Table 9) are plotted by the function of $1/T$, and they were fitted by the Arrhenius equation to extract the values of E_a: 26.0 ± 1.4 meV.

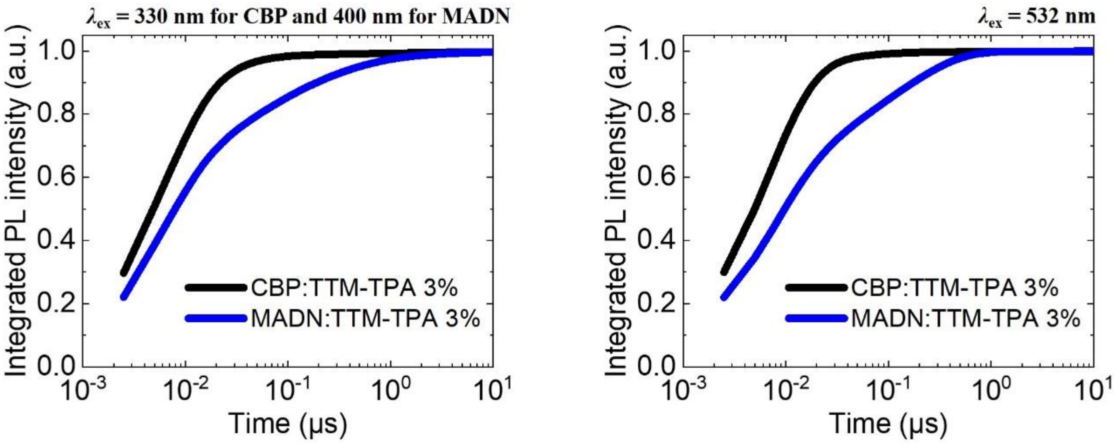

Supplementary Fig. 16 | The normalised integrated PL intensity of the CBP:TTM-TPA 3% and MADN:TTM-TPA 3% films with different excitation wavelengths.

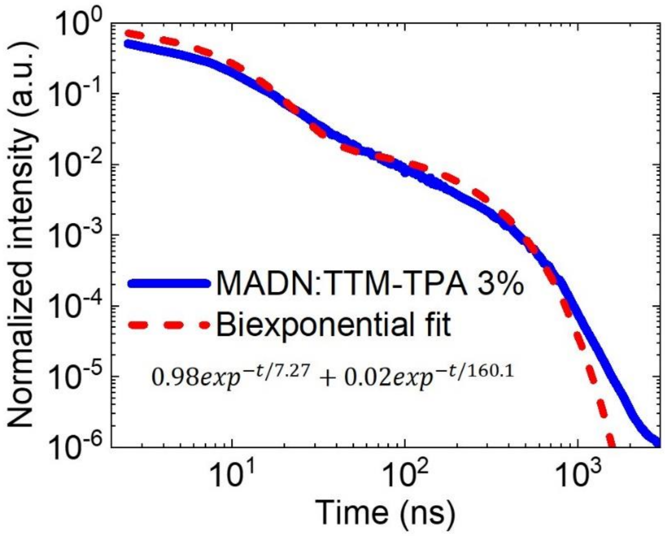

Supplementary Fig. 17 | The biexponential fit of the transient PL profile obtained by 532 nm excitation for the MADN:TTM-TPA 3% film.

Supplementary Table 7 | Summary of the intensity ratios, the efficiencies of total, prompt, and delayed emission, and the efficiency of R-DET in the MADN:TTM-TPA system.

Host	Dopant	r_1	r_2	Φ_{total}	Φ_{p}	Φ_{d}	$\Phi_{\mathrm{R-DET}}$
MADN	TTM-TPA	0.69	0.31	0.27	0.186	0.084	0.31

Supplementary Table 8 | Summary of the exciton decay rate and lifetime and DET rate constants by biexponential fit in the MADN:TTM-TPA system. The film was encapsulated and measured at room temperature in ambient condition.

Host	Dopant	τ_p (10^{-9} s)	τ_d (10^{-9} s)	k_p (10^7/s)	k_d (10^5/s)	$k_{r,D}$ (10^6/s)	$k_{nr,D}$ (10^6/s)	$k_{R\text{-}DET}$ (10^6/s)	$k_{F\text{-}DET}$ (10^5/s)
MADN	TTM-TPA	7.27	160.1	2.56	6.03	4.77	12.91	7.94	8.74

Supplementary Table 9 | Summary of the decay time (τ) and intensity ratio (W) at different temperatures by triexponential fit for MADN:TTM-TPA 3% excited at 532 nm. The film was measured without encapsulation in a cryostat under high vacuum (~10^{-5} mbar).

T (K)	τ_1 (ns)	W_1 (%)	τ_2 (μs)	W_2 (%)	τ_3 (μs)	W_3 (%)	τ_d (μs)
300	8.3	49	0.048	18	0.307	33	0.216
250	8.3	58	0.057	19	0.407	24	0.252
200	8.2	63	0.053	14	0.486	23	0.322
160	8.2	67	0.080	12	0.812	20	0.538
120	8.6	71	0.143	14	2.13	15	1.171
80	8.7	73	0.201	12	6.02	15	3.434
50	9.5	76	0.542	13	34.2	11	15.969
30	9.3	75	0.614	13	52.7	12	25.615
20	9.5	75	0.572	13	58.1	12	28.185
10	9.0	77	0.569	12	62.7	12	31.635
6	9.1	76	0.583	12	65.1	12	32.842

S4. Modelling of energy transfer

Single molecule calculations. The host MADN and radical TTM-TPA structures were optimised at the DFT level with the ωB97X-D functional and the 6-31G(d,p) basis set (we note that unrestricted Kohn-Sham (UKS) DFT was used for the open-shell molecule). In carrying out geometry optimisations (both for the fragments and the molecular complexes, see below) we opted for the ωB97X-D functional thanks to its demonstrated reliability in characterizing ground state geometries.[16] Yet, to improve the accuracy in the calculations of electronic properties, especially in computing vertical excitation energies, we chose to use the range-separated hybrid (RSH) LC-ωhPBE functional.[17] On each optimised molecular fragment, the range-separation parameter ω was gap-tuned when using the LC-ωhPBE/6-311G(d,p) level of theory, following the procedure described elsewhere.[18] For MADN an optimally-tuned (OT) ω value was found at 0.133 $Bohr^{-1}$, while for the radical TTM-TPA at 0.108 $Bohr^{-1}$. Time-dependent (TD) DFT calculations were then carried out on MADN and TTM-TPA, using the Tamm-Dancoff approximation (TDA),[19] resorting to an OT *screened* RSH (SRSH) approach and setting the macroscopic dielectric constant of toluene (2.37). By doing this, we obtained a qualitative description of the low-lying excited states: the first singlet, S_1, and triplet, T_1, for MADN and the first two doublets, D_1 and D_2, for TTM-TPA. Natural transition orbitals (NTOs) of these four excited states are shown in Supplementary Fig. 18. While S_1 and T_1 of MADN shows the same HOMO → LUMO transition, D_1 in TTM-TPA displays an intramolecular charge-transfer (*intra*-^{2}CT) character (*i.e.,* hole on the triphenylamine group and electron on the TTM core), while D_2 has a locally excited (LE) nature, being entirely localised on the TTM moiety. The excited states energies and NTOs of the fragments provide a reasonable proxy for those of the molecular host:emitter complexes (CPs), formed by MADN and TTM-TPA. All (TD)DFT calculations were performed using the GAUSSIAN16 suite,[20] except where otherwise stated.

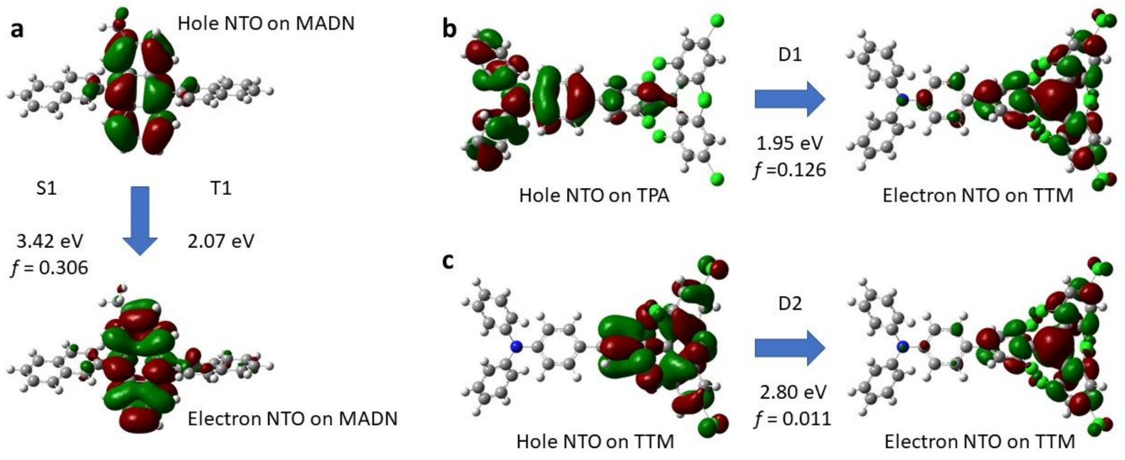

Supplementary Fig. 18 | Natural transition orbitals (NTOs) of the first singlet, S_1, and triplet, T_1, excited states of MADN (a), the first D_1 (b) and the second D_2 (c) doublet excited states of TTM-TPA. Vertical excitation energies and oscillator strengths are reported as well.

Sample Preparation. Classical Molecular Dynamics (MD) simulations were performed in order to build an amorphous MADN:TTM-TPA sample, made of 324 host MADN molecules doped with 6 radicals TTM-TPA in a m/m concentration of 3.1%. The general AMBER force field (GAFF)[21] for organic molecules was used, where atomic ESP charges were computed at the DFT ωB97X-D/6-311++G(d,p) level of theory on the previously optimised species.

An initial NVT simulation to introduce thermal disorder was run for 500 ps at 2000 K on a cubic box with each side of 100 Å and a starting density of ~0.2 g/cm^3. Then, a series of NPT simulations (7 ns in total) were run by compressing and relaxing the applied pressure from 1000 to 1 atm and by cooling the system from 1000 K down to 298 K. At last, a 5 ns NPT was run at 1 atm and 298 K, reaching a final density of 1.125 g/cm^3. At the end of the MD simulations, the final cell parameters were a = 60.6 Å, b = 60.4 Å and c = 59.9 Å while the angles α, β and γ were kept fixed at 90° (see Supplementary Fig. 19). All MD simulations were performed with the NAMD software,[22] using a timestep of 1 fs, 3D periodic boundary conditions and the particle mesh Ewald summation for electrostatic interactions, a cutoff of 12 Å for Lennard-Jones interactions, the velocity rescaling thermostat and the Berendsen barostat.

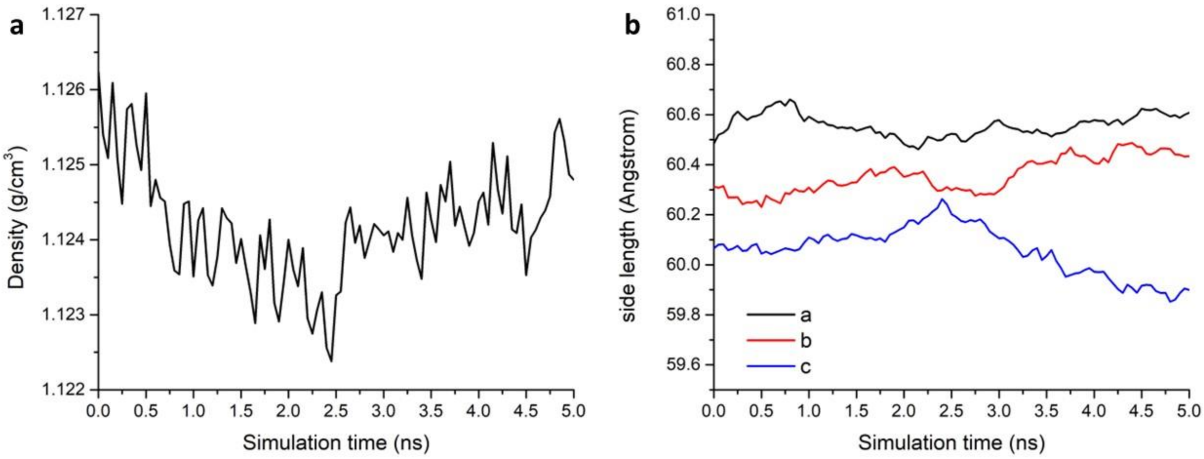

Supplementary Fig. 19 | Sample density (a) and cell side lengths (b) as a function of the simulation time for the NPT last 5 ns MD run.

The last MD frame (where each snapshot was taken every 50 ps) was used as a representative sample for the subsequent analysis of the excited state energetic landscape and electronic couplings of MADN:TTM-TPA CPs.

Molecular complexes calculations. Each MADN:TTM-TPA pair was selected according to geometrical criteria: a coarser selection on the molecular CP centre of mass distance, less than 12 Å, and a tighter one on the atom-atom distance, less than 4 Å. The structures of the selected CPs (36 in total) were further relaxed by means of UKS DFT ωB97X-D/6-31G(d,p). After the DFT optimisation, the pair labelled **CP1** was identified as the most stable in terms of ground state total energy. On that specific MADN:TTM-TPA pair, the ω value was gap-tuned at 0.095 Bohr^{-1} by using the LC-ωhPBE/6-311G(d,p) level of theory. Then, OT-SRSH UKS TDA TDDFT calculations were carried out in toluene on 17 CPs (the first 10 were selected according to the energy rank, while the other 7 were randomly chosen to scan other potential relative positions and orientations of MADN and TTM-TPA). The molecular CPs used in this work are shown in Supplementary Fig. 20. Results of the optimised **CP1** are discussed in the main text. In addition, constrained DFT (c-DFT) calculations, with charge restrictions imposed on the

constituting fragments of each molecular CP, were performed using the Q-Chem program package[23] and with the same level of theory as described above (OT-SRSH UKS TDA TDDFT in toluene). For each CP, the *inter*-^{2}CT state energy computed with c-DFT, before (from MD) and after geometry optimisation (from DFT optimisation) is reported in Supplementary Table 10, while the *inter*-^{2}CT NTOs of few selected CPs are shown in Supplementary Fig. 21.

Supplementary Table 10 | Energy of the lowest-lying *inter*-^{2}CT state computed using constrained DFT (c-DFT) from MD and DFT optimised structures of the selected 17 molecular complexes (CPs).

CP #	from MD [eV]	from DFT optimisation [eV]
**1**	2.85	2.56
**2**	2.61	2.25
**3**	2.63	2.47
**4**	2.08	1.62
**5**	2.41	1.53
**6**	2.53	1.49
**7**	3.55	2.21
**8**	2.44	1.53
**9**	2.14	2.06
**10**	3.46	2.98
**11**	2.21	1.82
**12**	2.38	2.66
**13**	1.86	1.79
**14**	3.23	3.09
**15**	1.95	1.62
**16**	2.98	1.80
**17**	2.51	1.29

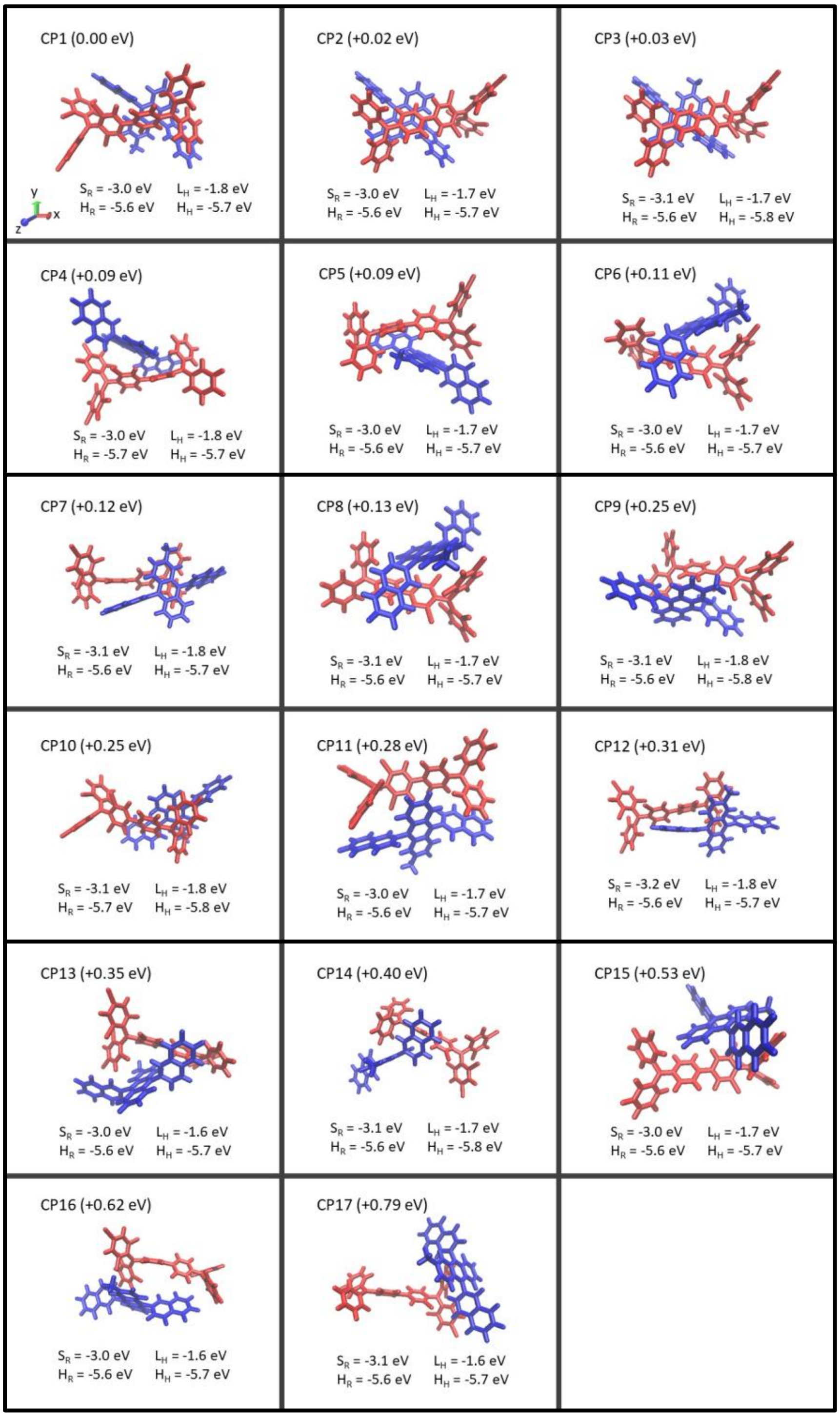

Supplementary Fig. 20 | The 17 molecular complexes (CPs) structures, showing the MADN host (H) in blue and the TTM-TPA radical (R) in red. After the UKS DFT optimisation performed with ωB97X-D/6-31G(d,p), these pairs were ranked in terms of ground state total energy with respect to CP1 (*i.e.*, the most stable pair) and their relative stability is given in parentheses. Frontier molecular orbital energies, computed at the OT-SRSH LC-ωhPBE/6-311G(d,p) level in toluene, are also reported: S_R and H_R refer to the SOMO and HOMO of the TTM-TPA radical, respectively, while L_H and H_H to the LUMO and HOMO of the MADN host, respectively.

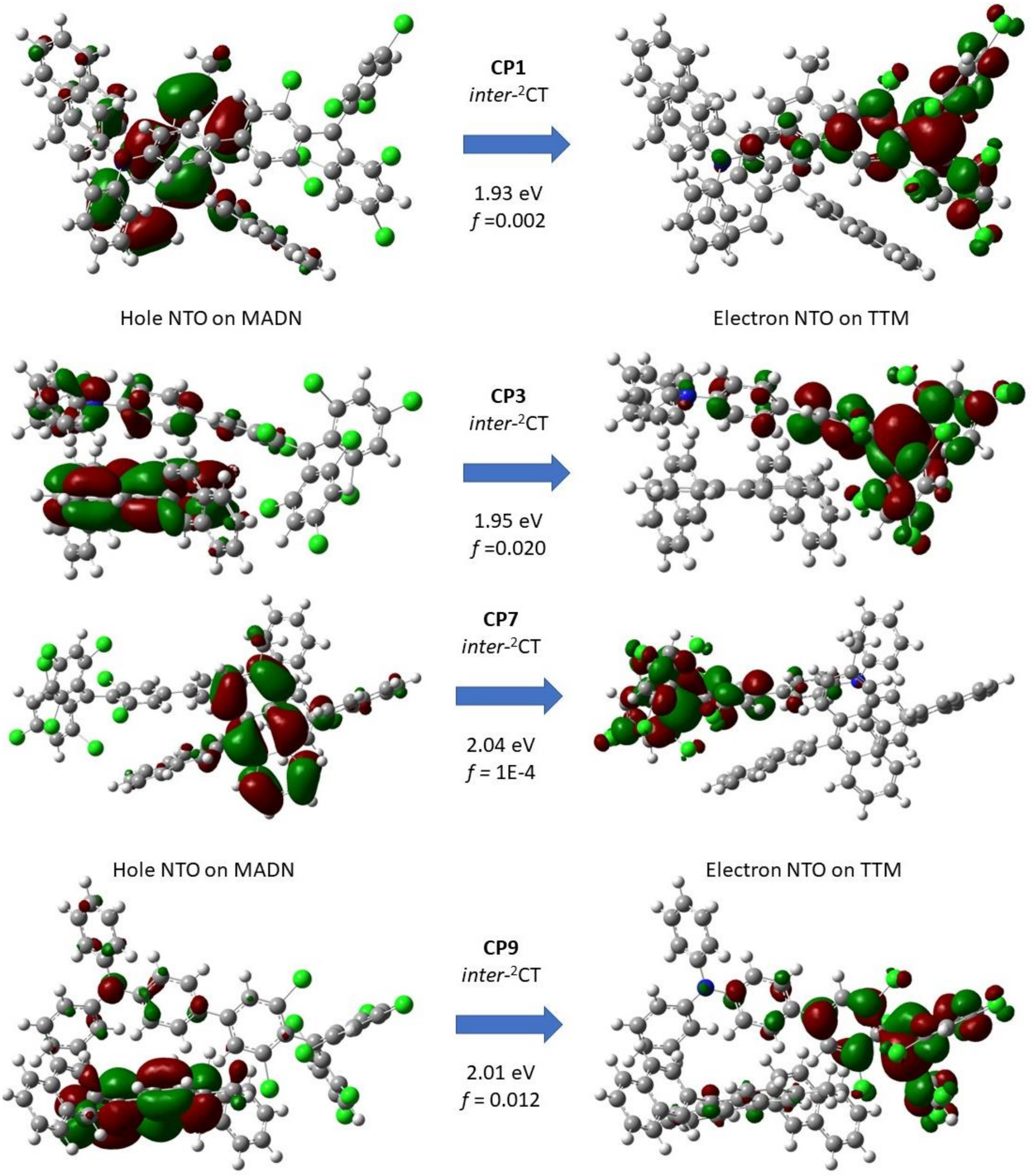

Supplementary Fig. 21 | Natural transition orbitals (NTOs) representing the lowest-lying *inter*-^{2}CT state for few selected molecular complexes. Vertical excitation energies and oscillator strengths are also given.

Calculation of excitation energy transfer (EET) rates. The Marcus-Levich-Jortner (MLJ) equation, used to compute EET rates, reads as follow:

$$\kappa_{EET} = \frac{2\pi}{\hbar} V_{EET}^2 \sqrt{\frac{1}{4\pi\lambda_s k_B T}} \times$$

$$\times \sum_n \left\{ \exp(-S_{eff}) \frac{S_{eff}^n}{n!} \times exp\left[-\frac{\left(-\Delta E_{S(T)1-D1(2)}^0 + \lambda_s + n\hbar\omega_{eff}\right)^2}{4\lambda_s k_B T} \right] \right\} \quad \text{(S23)}$$

where $\Delta E_{S(T)1-D1(2)}^0$ is the energy difference between two excited states (either S_1 or T_1 in MADN and D_1 or D_2 in TTM-TPA) and in this work was taken from OT-SRSH UKS TDA TDDFT calculations, V_{EET} is the electronic coupling associated to the EET process (see below), λ_s is the external reorganisation energy, S_{eff} is the Huang-Rhys factor describing the coupling of the energy transfer to an effective, internal normal mode of frequency ω_{eff}, where S_{eff} was obtained directly from the internal reorganisation energy λ_i as $S_{eff} = {}^{\lambda_i}/_{\hbar\omega_{\mathrm{eff}}}$.

Calculation of reorganisation energies. To compute internal (λ_i) and external (λ_s) reorganisation energies contributions of both the host MADN and radical TTM-TPA to the different EET processes, we used a displaced harmonic oscillator model. In this model, each intramolecular normal mode is projected on the vector describing the structural changes between the optimised ground state geometry and the excited state one, thereby partitioning the reorganisation energy into mode contributions. A vibrational analysis was carried out both for the optimised S_0 ground state, S_1 and T_1 excited state of MADN and for the optimised D_0 ground state, D_1 and D_2 excited states of TTM-TPA. Ground state geometry optimisation on fragments were performed as previously described, while excited state optimisations were carried out at the UKS TDA TDDFT ωB97X-D/6-31G(d,p) level of theory. All the frequencies

were computed and checked to be positive, and the normal mode decomposition to the reorganisation energy was carried out with the MOMAP software.[24]

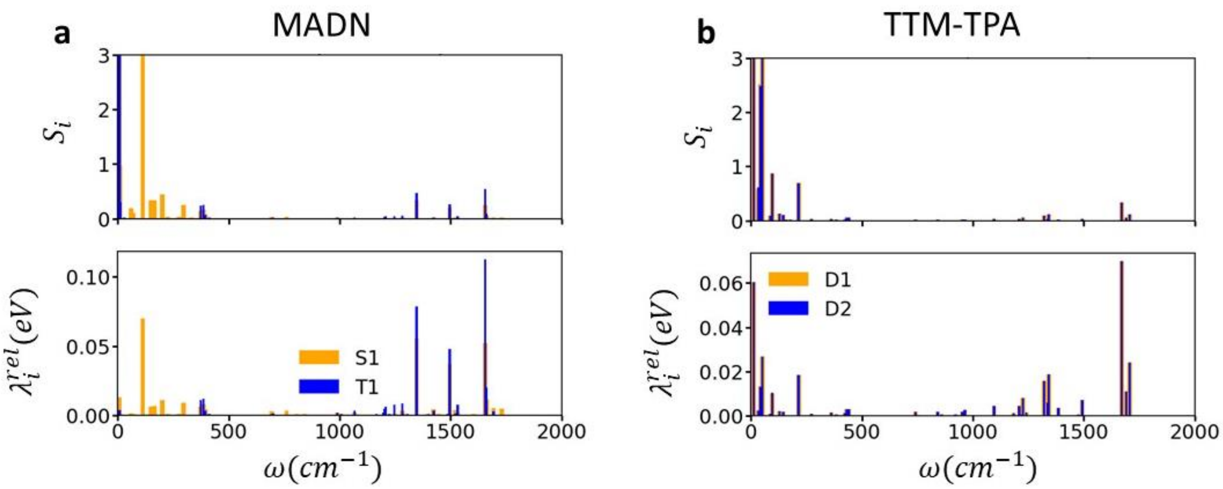

Supplementary Fig. 22 | Huang-Rhys factor for each computed normal mode that is coupled to the excitation and its relative weight to the total reorganisation energy for (a) S_1 and T_1 of MADN and (b) D_1 and D_2 of TTM-TPA.

By applying a threshold at 1000 cm^{-1}, we distinguished between the intramolecular part of the reorganisation energy, λ_i, given by the high-frequency, quantum modes (above the threshold), and the intermolecular part, λ_s, given by the low-frequency, classic modes (below the threshold). From Supplementary Fig. 22, if one the one hand, low-frequency vibrations strongly couple to the reorganisation energy of S_1 in MADN and the high-frequency modes to that of T_1 (panel **a**), on the other hand, D_1 and D_2 in TTM-TPA show a very similar normal mode decomposition (panel **b**). Therefore, we can assume that D_1 and D_2 have the same λ_i and λ_s parameters (see Supplementary Table 11). The contributions to the various EET processes in the molecular CPs were computed as:

$$\lambda_i^{S(T)1-D1(2)} = \lambda_i^{S(T)1} + \lambda_i^{D1(2)} \quad \text{(S24)}$$

$$\lambda_s^{S(T)1-D1(2)} = \lambda_s^{S(T)1} + \lambda_s^{D1(2)} \quad \text{(S25)}$$

The effective frequency $\hbar\omega_{eff}$ entering the MLJ equation was computed by a weighted average over the high-frequency modes as: $\hbar\omega_{eff} = \frac{\sum_i \hbar\omega_i S_i}{\sum_i S_i}$.

Supplementary Table 11 | Reorganisation energies and related parameters for S_1-D_1(2) and T_1-D_1 excitation energy transfer (EET) processes.

	S_1-D_1(2)	T_1-D_1
λ_i (eV)	0.378	0.501
λ_s (eV)	0.324	0.205
$\hbar\omega_{eff}$ (eV)	0.185	0.185
S_{eff}	2.046	2.717

Calculation of electronic couplings. The electronic coupling between two interacting molecules can be computed, within a 1st-order perturbative approximation, directly from the transition densities of the noninteracting fragments. This scheme, called direct coupling (DC), was developed in the TDDFT framework under the assumption that the molecular orbitals can be well separated into two fragments.[25] In such scheme the coupling can be written as:

$$V_{DC} = V_{Coul} + V_{xc} + V_{ovl} =$$

$$= \int d\boldsymbol{r}d\boldsymbol{r}' \rho_D^{tr*}(\boldsymbol{r}) \frac{1}{|r-r'|} \rho_A^{tr}(\boldsymbol{r}') - \int d\boldsymbol{r}d\boldsymbol{r}' \rho_D^{tr*}(\boldsymbol{r}) g_{xc}(\boldsymbol{r},\boldsymbol{r}') \rho_A^{tr}(\boldsymbol{r}') - \omega_0 \int d\boldsymbol{r} \rho_D^{tr*}(\boldsymbol{r}) \rho_A^{tr}(\boldsymbol{r}) \quad \text{(S26)}$$

where ρ_D is the transition density of the donor fragment, corresponding to the S_1 excited state localised on the host MADN, while ρ_A is the transition density related to the D_1 (or D_2) excited states of TTM-TPA, ω_0 is the average transition energy and g_{xc} is the exchange-correlation kernel given by the used functional. Here, electronic couplings were computed with OT-SRSH UKS TDA TDDFT calculations in gas-phase at the LC-ωhPBE/6-311G(d,p) level of theory.

The three terms on the right-hand side of Eq. S26 represent the Coulomb, the exchange-correlation, and the overlap contribution to the whole coupling, respectively. In modelling the EET between S_1 of MADN and D_1 (or D_2) of TTM-TPA, the long-range Coulomb term V_{Coul} amounts to ~99% of the full coupling for all the chosen molecular CPs (see Supplementary Table 12 and 13).

Supplementary Table 12 | Energy difference (in eV) between S_1 of MADN and D_1 of TTM-TPA, the corresponding Coulomb coupling (in meV), the rate of the excitation energy transfer (EET) process (expressed in 10^9 s^{-1}) and its lifetime (in ps) for the selected 17 molecular complexes (CPs) (outliers are indicated with an asterisk).

CP #	ΔE^0_{S1-D1} [eV]	V_{Coul} [meV]	κ_{EET} [x10^9 s^{-1}]	τ_{EET} (ps)
**1**	1.33	-14.61	339	3.0
**2**	1.35	9.47	125	8.0
**3**	1.39	-11.72	154	6.5
**4**	1.21	-12.86	466	2.1
**5**	1.23	16.93	731	1.4
**6**	1.30	10.13	188	5.3
**7**	1.38	9.33	102	9.8
**8**	1.29	9.48	169	5.9
**9**	1.45	9.80	78.8	12.7
**10**	1.28	4.67	45.0	22.2
**11**	1.29	18.23	647	1.5
**12**	1.49	9.63	60.4	16.6
**13**	1.29	-12.56	304	3.3
**14**	1.47	-2.56	4.6	215*
**15**	1.24	6.02	88.4	11.3
**16**	1.17	-15.25	777	1.3
**17**	1.29	4.03	32.1	31.2

Supplementary Table 13 | Energy difference (in eV) between S_1 of MADN and D_2 of TTM-TPA, the corresponding Coulomb coupling (in meV), the rate of the excitation energy transfer (EET) process (expressed in 10^9 s^{-1}) and its lifetime (in ps) for the selected 17 molecular complexes (CPs) (outliers are indicated with an asterisk).

CP #	ΔE^0_{S1-D2} [eV]	V_{Coul} [meV]	κ_{EET} [x10^9 s^{-1}]	τ_{EET} (ps)
**1**	0.35	2.57	51.3	19.5
**2**	0.50	-0.01	1.5x10^{-3}	6.5x10^5*
**3**	0.50	1.91	43.6	23.0
**4**	0.43	-2.61	69.5	14.4
**5**	0.43	-2.69	74.7	13.4
**6**	0.46	-0.32	1.2	865*
**7**	0.48	-2.57	76.1	13.1
**8**	0.40	0.04	0.01	7.8x10^4*
**9**	0.52	1.07	14.1	70.7
**10**	0.40	-2.66	65.0	15.4
**11**	0.46	-1.69	31.2	32.0
**12**	0.46	-4.55	231	4.3
**13**	0.48	3.87	173	5.8
**14**	0.56	1.64	34.6	28.9
**15**	0.43	-5.90	356	2.8
**16**	0.45	1.14	13.8	72.3
**17**	0.52	7.97	786	1.3

In contrast, the Coulomb contribution V_{Coul} is exactly zero when we consider the EET between T_1 of MADN and D_1 of TTM-TPA, since T_1 has null transition density. In this case, V_{xc} and V_{ovl} in Eq. S26 become dominant. Moreover, low-lying *inter*-^{2}CT states, in close energy resonance with T_1 and D_1, might substantially mix with the D_1 state on TTM-TPA (which has

an *intra*-^{2}CT state nature) and potentially participate as mediating states in the triplet-doublet energy transfer. To describe this EET in a superexchange-mediated (SE) mechanism, we computed the electronic coupling related to this process by using a perturbative treatment as:

$$V_{SE} = V_0 + V_1 = V_0 + \frac{V_{T1-CT}\, V_{CT-D1}}{\frac{(E_{T1} + E_{D1})}{2} - E_{CT}} \qquad \text{(S27)}$$

where E_{T1}, E_{D1} and E_{CT} are the excitation energies taken from OT-SRSH UKS TDA TDDFT calculations, $V_0 = V_{xc} + V_{ovl}$ is the 1st-order term obtained as previously described, V_{T1-CT} is the coupling between the initial T_1 of MADN and the *inter*-^{2}CT state, V_{CT-D1} between the *inter*-^{2}CT state and the final D_1 of TTM-TPA. These values were approximated in this work by using one-electron transfer integrals involving the frontier molecular orbitals of the single fragments and describing the relative electron transfer:

$$V_{T1-CT} = \langle \varphi_{LUMO}(MADN) | \hat{H} | \varphi_{SOMO}(TTM - TPA) \rangle \qquad \text{(S28)}$$

$$V_{CT-D1} = \langle \varphi_{HOMO}(TTM - TPA) | \hat{H} | \varphi_{HOMO}(MADN) \rangle \qquad \text{(S29)}$$

where the first equation represents the photoinduced electron transfer (PET) from the LUMO of MADN to the SOMO of TTM-TPA, the second one the PET from the HOMO of MADN and the HOMO of TTM-TPA and where $\hat{H}$ is the Kohn-Sham operator of the interacting molecular CP. The rates related to this Dexter-like process were obtained with MLJ equation and are reported in Supplementary Table 14. We also note that, out of 17 pairs, we discarded 6 of them, as in those CPs the *inter*-^{2}CT and D1 states heavily mix, and thus affecting their excitation energy values.

Supplementary Table 14 | Energy difference (in eV) between T_1 of MADN and D_1 of TTM-TPA, the one-electron transfer integrals, V_0 and V_1 (in meV), the rate of the excitation energy transfer (EET) process (expressed in 10^6 s^{-1}) and its lifetime (in ns) considering as coupling $V_{SE} = V_0 + V_1$ (outliers are indicated with an asterisk).

CP #	ΔE^0_{T1-D1} [eV]	V_{T1-CT} [meV]	V_{CT-D1} [meV]	V_0 [meV]	V_1 [meV]	κ_{EET} [x10^6 s^{-1}]	τ_{EET} (ns)
**1**	0.16	6.51	0.74	0.12	-0.79	1.3x10^3	0.77
**3**	0.15	7.72	33.60	0.08	32.62	2.8x10^6	3.6x10^{-4}*
**7**	0.16	1.33	21.49	0.09	-0.23	55.1	18.2
**9**	0.17	4.13	105.94	0.16	-5.87	1.0x10^5	0.01
**10**	0.09	11.73	1.92	0.04	-0.17	23.2	43.1
**12**	0.25	12.56	10.16	0.04	-1.54	1.1x10^4	0.09
**13**	0.02	5.00	13.74	0.13	0.33	103	9.7
**14**	0.18	0.02	76.59	0.01	-0.01	0.07	1.3x10^4*
**15**	0.02	47.18	19.13	0.05	4.30	9.4x10^3	0.11
**16**	-0.04	7.65	0.40	0.11	0.01	1.9	529*
**17**	0.05	32.81	5.80	0.03	0.66	365	2.7

Triplet exciton diffusion. In the effort to quantify the diffusion rates of molecular triplets localised on the host matrix, we built three different clusters (A, B and C) made of MADN dimers (*i.e.*, a cluster centered on a randomly selected MADN plus its first shell of solvation). Then, we carried out OT-SRSH TDA TDDFT calculations in gas-phase at the LC-ωhPBE/6-311G(d,p) level of theory and we computed the electronic couplings for triplets of all the nearest MADN dimers by applying a multi-state diabatization procedure.[26,27] The corresponding triplet energy transfer (TET) rates were then computed by using the MLJ equation in Eq. S23, where the energy difference ΔE^0_T between the initial and final triplet excited state was set to zero and the reorganisation energies were computed as explained above.

For the triplet excitation transfer from a single MADN molecule to another one, λ_i = 0.642 eV, λ_s = 0.089 eV, $\hbar\omega_{\text{eff}}$ = 0.183 eV and S_{eff} = 3.515. Results for the three different clusters are reported in Supplementary Table 15. As expected, TET couplings are extremely small, and the TET rates span over multiple orders of magnitude. Nonetheless, the fastest events occur in the range of 3-24 ns.

Supplementary Table 15 | Triplet energy transfer (TET) couplings (absolute value, in meV), the rate of the TET process (expressed in s^{-1}) and its lifetime (in s). For each cluster (A, B and C), the 1st MADN is the molecule at the centre of the cluster, while the 2nd one is included in the first shell of solvation of the 1st MADN.

1st MADN	2nd MADN	$\|\|V_{TET}\|\|$ [meV]	κ_{TET} [s^{-1}]	τ_{TET} (s)
A1	A2	0.008	$4.3x10^{4}$	$2.3x10^{-5}$
A1	B1	0.304	$6.6x10^{7}$	$1.5x10^{-8}$
A1	A3	0.001	$6.8x10^{2}$	$1.5x10^{-3}$
A1	A4	0.003	$6.6x10^{3}$	$1.5x10^{-4}$
A1	A5	0.042	$1.3x10^{6}$	$7.9x10^{-7}$
A1	C1	0.028	$5.5x10^{5}$	$1.8x10^{-6}$
A1	A6	0.404	$1.2x10^{8}$	$8.6x10^{-9}$
A1	A7	0.001	$1.2x10^{3}$	$8.6x10^{-4}$
B1	A2	0.242	$4.2x10^{7}$	$2.4x10^{-8}$
B1	A3	0.008	$4.1x10^{4}$	$2.4x10^{-5}$
B1	A4	0.004	$1.4x10^{4}$	$7.1x10^{-5}$
B1	B2	0.005	$1.5x10^{4}$	$6.6x10^{-5}$
B1	B3	0.288	$5.9x10^{7}$	$1.7x10^{-8}$
B1	A5	0.075	$4.0x10^{6}$	$2.5x10^{-7}$
B1	A6	0.003	$5.2x10^{3}$	$1.9x10^{-4}$
C1	C2	0.017	$2.0x10^{5}$	$5.1x10^{-6}$
C1	C3	0.011	$8.3x10^{4}$	$1.2x10^{-5}$
C1	C4	0.069	$3.4x10^{6}$	$2.9x10^{-7}$
C1	C5	0.015	$1.6x10^{5}$	$6.1x10^{-6}$
C1	C6	0.039	$1.1x10^{6}$	$9.2x10^{-7}$
C1	A6	0.019	$2.5x10^{5}$	$3.9x10^{-6}$
C1	A7	0.704	$3.5x10^{8}$	$2.8x10^{-9}$

S5. Angle-dependent PL measurement

The angle-dependent PL measurement was conducted using a rotational stage, a half-cylindrical lens, and a polariser with 400 nm laser excitation to characterise the internal propagation angle distribution of photons in the substrate. PL spectra were recorded from 0° to 90° by an Andor spectrometer (Shamrock 303i) with an Andor iDus CCD array. We have fitted the measured angular distribution with a transfer-matrix formalism (TMF) calculation by varying the ratio between the vertical and horizontal dipoles.[28,29] The best fitting was achieved with the horizontal dipole ratio of 85% for the MADN:TTM-TPA system (Supplementary Fig. 23). Such a strong anisotropy and horizontal alignment of dipoles are highly beneficial for the outcoupling of photons in OLEDs.

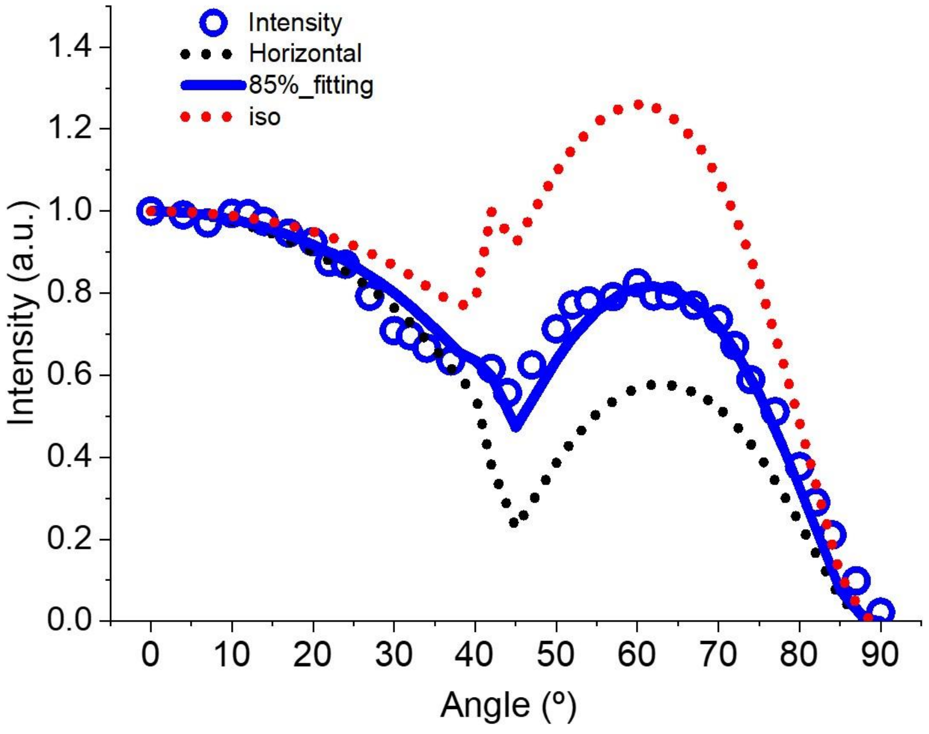

Supplementary Fig. 23 | The angle-dependent PL measurement for TTM-TPA 3 wt% doped in MADN.

S6. Cyclic voltammetry

The cyclic voltammetry (CV) measurements on TTM-TPA were performed using a CHI660E electrochemical analyzer with a glass carbon disk as the working electrode, a platinum wire as the counter electrode, and Ag/Ag+ as the reference electrode. TTM-TPA is dissolved in dichloromethane (DCM) at 1 mM, a sweep rate of 50 mV s^{-1} was utilised, and the ferrocenium/ferrocene redox couple was used as an internal standard. Consecutive 20-cycle experiment is shown in Supplementary Fig. 24, showing redox stability under these conditions with oxidation and reduction peaks. From the cyclic voltammogram shown in Supplementary Fig. 24, the TTM-TPA SOMO energy level (reduction) is calculated from CV potentials using the HOMO of ferrocene (−4.8 eV) as the standard: TTM-TPA SOMO = −4.8 + ($-E_{1/2}^{red}$: 1.0) = −3.8 eV and HOMO is determined as SOMO – E_g^{opt} (onset of the UV-Vis spectrum in toluene). Additionally, the energy levels of TTM-TPA, TTM-1Cz, and TTM-3PCz are compared in Supplementary Table 16.

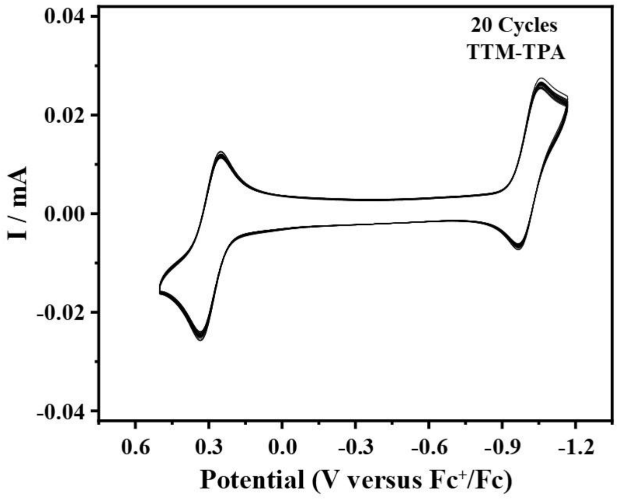

Supplementary Fig. 24 | Multi-cycle CV measurements (20 cycles) of TTM-TPA.

Supplementary Table 16 | Electronic and optical properties of TTM-TPA compared to other radicals. Frontier energy levels of TTM-TPA vs TTM-1Cz and TTM-3PCz as obtained by cyclic voltammetry method. PL peak and PLQE for different radicals in toluene solution.

	HOMO (eV)	SOMO (eV)	PL peak (nm)	PLQE (%)
TTM-1Cz	-5.9	-3.9	670	41
TTM-3PCz	-5.7	-3.8	695	46
TTM-TPA	-5.4	-3.8	800	24

S7. Photostability

Firstly, the photostability experiment for TTM-TPA diluted in toluene (200 µM) was conducted under continuous wave UV illumination in FLS980 with a monochromated xenon arc lamp at λ_{Ex} = 400 nm. Photoluminescence intensity decay was not observed over a three-hour measurement window (Supplementary Fig. 25). Next, the photostability was measured under pulsed (200 fs) UV illumination at an extremely high fluence (2800 µJ cm^{-2}). A decay of only ~10% over one hour was recorded (Supplementary Fig. 26), which is comparable to that of TTM-1Cz, a well-studied stable luminescent radical. This excitation power is orders of magnitude larger than that used in the transient absorption and transient photoluminescence measurements and confirms the stability of the material during the relevant experiments.

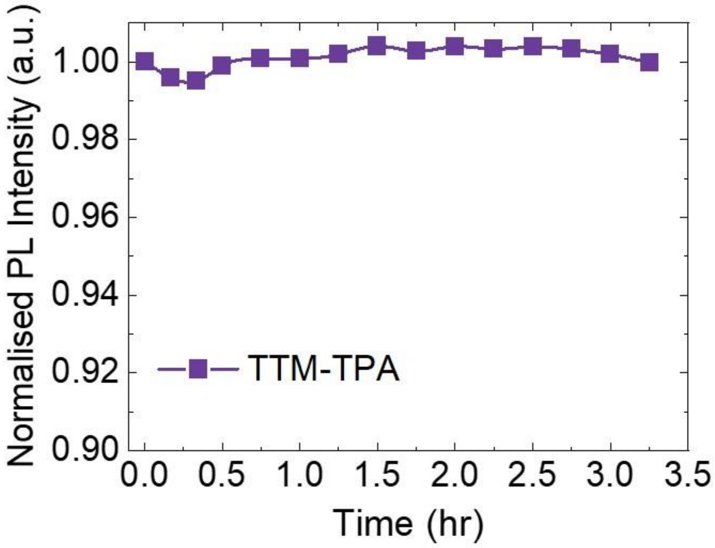

Supplementary Fig. 25 | Photostability of TTM-TPA with continuous photoexcitation at 400 nm.

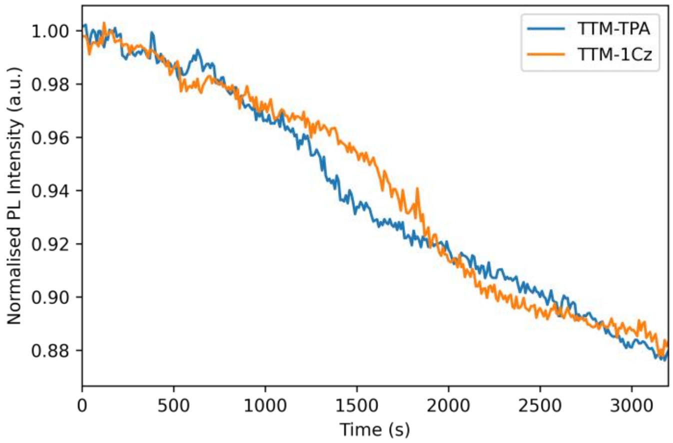

Supplementary Fig. 26 | Photostability of TTM-TPA and TTM-1Cz with pulsed (200 fs) laser excitation of 400 nm at a fluence of 2800 µJ cm^{-2}.

S8. Ultraviolet photoelectron spectroscopy

For the ultraviolet photoelectron spectroscopy (UPS), 50 nm-thick TTM-TPA film was deposited on an ITO substrate in a vacuum chamber (~10^{-7} torr). The sample was loaded into a transfer vessel in a glove box and transferred to a nitrogen chamber. UPS measurements were carried out in a UHV chamber of a photoelectron spectroscopy system (Thermo Scientific ESCALAB 250Xi) and using a double-differentially pumped He discharge lamp (hν = 21.22 eV) with a pass energy of 2 eV and a bias at −4 V. Supplementary Fig. 27 shows the UPS data plot showing the HOMO of TTM-TPA is 5.42 eV, which is comparable to the CV result above.

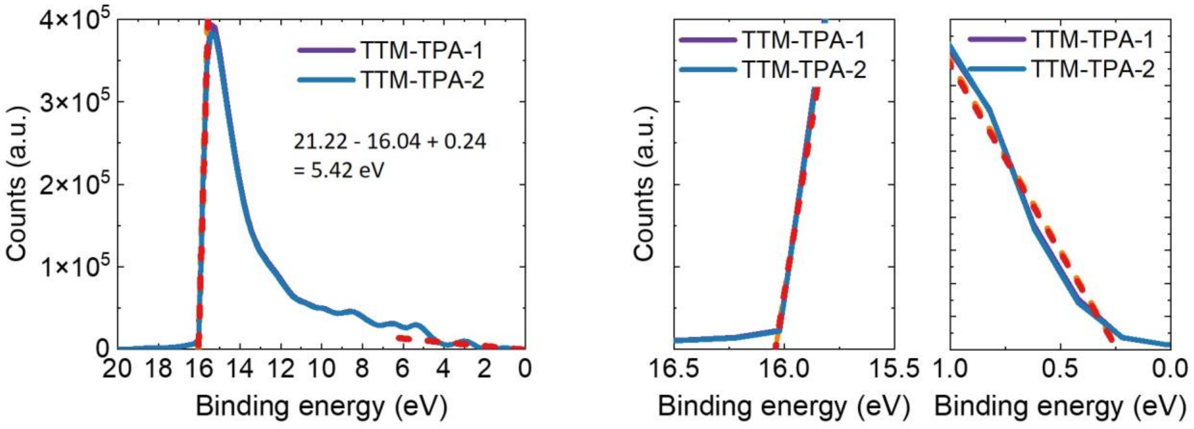

Supplementary Fig. 27 | Ultraviolet photoelectron spectroscopy (UPS) spectra for TTM-TPA.

S9. References

1. Murto, P. *et al.* Triazolobenzothiadiazole-Based Copolymers for Polymer Light-Emitting Diodes: Pure Near-Infrared Emission via Optimized Energy and Charge Transfer. *Adv. Opt. Mater.* **4**, 2068–2076 (2016).

2. Minotto, A. *et al.* Efficient near-infrared electroluminescence at 840 nm with "Metal-free" small-molecule:Polymer blends. *Adv. Mater.* **30**, 1706584 (2018).

3. Graham, K. R. *et al.* Extended conjugation platinum(II) porphyrins for use in near-infrared emitting organic light emitting diodes. *Chem. Mater.* **23**, 5305–5312 (2011).

4. Xue, J. *et al.* Homoleptic Facial Ir(III) Complexes via Facile Synthesis for High-Efficiency and Low-Roll-Off Near-Infrared Organic Light-Emitting Diodes over 750 nm. *Chem. Mater.* **29**, 4775–4782 (2017).

5. Sommer, J. R. *et al.* Efficient near-infrared polymer and organic light-emitting diodes based on electrophosphorescence from (tetraphenyltetranaphtho[2,3]porphyrin)platinum(II). *ACS Appl. Mater. Interfaces* **1**, 274–278 (2009).

6. Wei, Y. C. *et al.* Overcoming the energy gap law in near-infrared OLEDs by exciton–vibration decoupling. *Nat. Photonics* **14**, 570–577 (2020).

7. Kim, D. H. *et al.* High-efficiency electroluminescence and amplified spontaneous emission from a thermally activated delayed fluorescent near-infrared emitter. *Nat. Photonics* **12**, 98–104 (2018).

8. Brodeur, J. *et al.* Highly Efficient and Spectrally Narrow Near-Infrared Fluorescent OLEDs Using a TADF-Sensitized Cyanine Dye. *Adv. Opt. Mater.* **7**, 1901144 (2019).

9. Shahalizad, A. *et al.* Efficient Solution-Processed Hyperfluorescent OLEDs with Spectrally Narrow Emission at 840 nm. *Adv. Funct. Mater.* **31**, 2007119 (2021).

10. Liu, T., Xie, G., Zhong, C., Gong, S. & Yang, C. Boosting the Efficiency of Near-Infrared Fluorescent OLEDs with an Electroluminescent Peak of Nearly 800 nm by Sensitizer-Based Cascade Energy Transfer. *Adv. Funct. Mater.* **28**, 1706088 (2018).

11. Cho, H. H. *et al.* Near-Infrared Light-Emitting Diodes from Organic Radicals with Charge Control. *Adv. Opt. Mater.* **10**, 2200628 (2022).

12. Valeur, B. *Molecular fluorescence : principles and applications*. (Wiley-VCH, 2001).

13. Baldo, M. A. & Forrest, S. R. Transient analysis of organic electrophosphorescence: I. Transient analysis of triplet energy transfer. *Phys. Rev. B* **62**, 10958–10966 (2000).

14. Goushi, K., Yoshida, K., Sato, K. & Adachi, C. Organic light-emitting diodes employing efficient reverse intersystem crossing for triplet-to-singlet state conversion. *Nat. Photonics* **6**, 253–258 (2012).

15. Tao, Y. *et al.* Thermally activated delayed fluorescence materials towards the breakthrough of organoelectronics. *Adv. Mater.* **26**, 7931–7958 (2014).

16. Mardirossian, N. & Head-Gordon, M. Thirty years of density functional theory in computational chemistry: An overview and extensive assessment of 200 density functionals. *Mol. Phys.* **115** 2315–2372 (2017).

17. Cho, E., Coropceanu, V. & Brédas, J. L. Organic Neutral Radical Emitters: Impact of Chemical Substitution and Electronic-State Hybridization on the Luminescence Properties. *J. Am. Chem. Soc.* **142**, 17782–17786 (2020).

18. Gillett, A. J. *et al.* Spontaneous exciton dissociation enables spin state interconversion in delayed fluorescence organic semiconductors. *Nat. Commun.* **12**, 6640 (2021).

19. Hirata, S. & Head-Gordon, M. *Time-dependent density functional theory within the Tamm-Dancoff approximation*. *Chem. Phys. Lett.* **314,** 291-299 (1999).

20. M. J. Frisch *et al.* Gaussian 16, Revision A.03.

21. Wang, J., Wolf, R. M., Caldwell, J. W., Kollman, P. A. & Case, D. A. Development and testing of a general Amber force field. *J. Comput. Chem.* **25**, 1157–1174 (2004).

22. Phillips, J. C. *et al.* Scalable molecular dynamics with NAMD. *J. Comput. Chem.* **26** 1781–1802 (2005).

23. Epifanovsky, E. et al. Software for the frontiers of quantum chemistry: An overview of developments in the Q-Chem 5 package. *J. Chem. Phys.* **155**, 084801 (2021).

24. Shuai, Z. Thermal Vibration Correlation Function Formalism for Molecular Excited State Decay Rates. *Chin. J. Chem.* **38**, 1223–1232 (2020).

25. Hsu, C. P., Fleming, G. R., Head-Gordon, M. & Head-Gordon, T. Excitation energy transfer in condensed media. *J. Chem. Phys.* **114**, 3065–3072 (2001).

26. Hsu, C. P. The electronic couplings in electron transfer and excitation energy transfer. *Acc Chem. Res.* **42**, 509–518 (2009).

27. Cupellini, L., Corbella, M., Mennucci, B. & Curutchet, C. Electronic energy transfer in biomacromolecules. *WIREs Comput. Mol. Sci.* **9***,* e1392 (2018).

28. Kim, K. H. & Kim, J. J. Origin and Control of Orientation of Phosphorescent and TADF Dyes for High-Efficiency OLEDs. *Adv. Mater.* **30**, 1705600 (2018).

29. Cho, C. & Greenham, N. C. Computational Study of Dipole Radiation in Re-Absorbing Perovskite Semiconductors for Optoelectronics. *Adv. Sci.* **8**, 2003559 (2021).
